# Mechanosensitive membrane domains regulate calcium entry in arterial endothelial cells to protect against inflammation

**DOI:** 10.1172/JCI175057

**Published:** 2024-05-21

**Authors:** Soon-Gook Hong, Julianne W. Ashby, John P. Kennelly, Meigan Wu, Michelle Steel, Eesha Chattopadhyay, Rob Foreman, Peter Tontonoz, Elizabeth J. Tarling, Patric Turowski, Marcus Gallagher-Jones, Julia J. Mack

**Affiliations:** 1 Department of Medicine, Division of Cardiology,; 2 Molecular Biology Institute,; 3 Department of Pathology and Laboratory Medicine, and; 4 Institute for Quantitative and Computational Biosciences, UCLA, Los Angeles, California, USA.; 5 UCL Institute of Ophthalmology, University College London, London, United Kingdom.; 6 Correlated Imaging, Rosalind Franklin Institute, Harwell Science & Innovation Campus, Didcot, United Kingdom.

**Keywords:** Cell biology, Vascular biology, Calcium signaling, Endothelial cells

## Abstract

Endothelial cells (ECs) in the descending aorta are exposed to high laminar shear stress, and this supports an antiinflammatory phenotype. High laminar shear stress also induces flow-aligned cell elongation and front-rear polarity, but whether these are required for the antiinflammatory phenotype is unclear. Here, we showed that caveolin-1–rich microdomains polarize to the downstream end of ECs that are exposed to continuous high laminar flow. These microdomains were characterized by high membrane rigidity, filamentous actin (F-actin), and raft-associated lipids. Transient receptor potential vanilloid (TRPV4) ion channels were ubiquitously expressed on the plasma membrane but mediated localized Ca^2+^ entry only at these microdomains where they physically interacted with clustered caveolin-1. These focal Ca^2+^ bursts activated endothelial nitric oxide synthase within the confines of these domains. Importantly, we found that signaling at these domains required both cell body elongation and sustained flow. Finally, TRPV4 signaling at these domains was necessary and sufficient to suppress inflammatory gene expression and exogenous activation of TRPV4 channels ameliorated the inflammatory response to stimuli both in vitro and in vivo. Our work revealed a polarized mechanosensitive signaling hub in arterial ECs that dampened inflammatory gene expression and promoted cell resilience.

## Abstract 2

Laminar blood flow compartmentalizes and activates signaling in arterial endothelial cells to promote cell resilience and an antiinflammatory phenotype via surface asymmetry.

## Graphical abstract

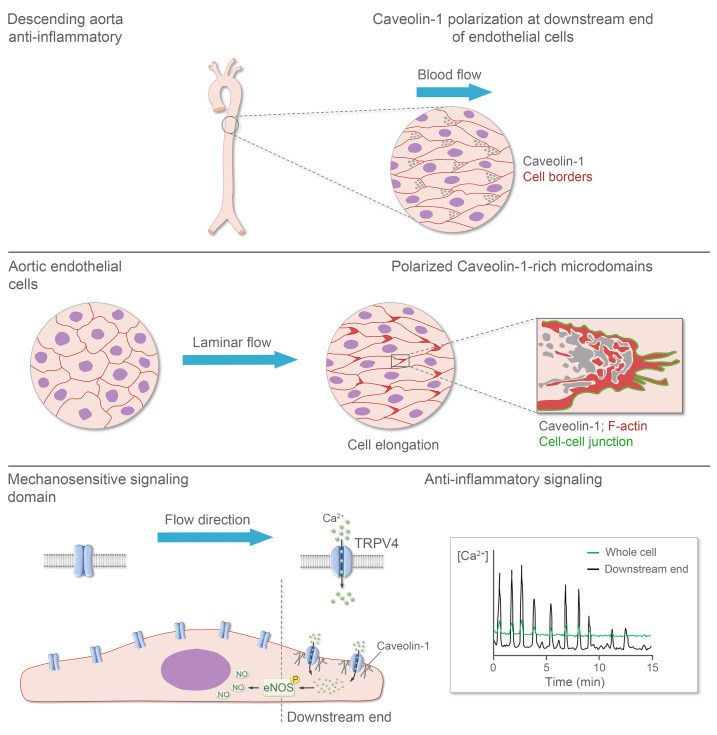

## Introduction

Blood flow patterns in the aorta are defined by vessel geometry: the curvature of the aortic arch results in low/oscillatory flow, whereas the straight descending aorta experiences high laminar flow (1, 2). The luminal layer of endothelial cells (ECs) in these two regions shows a striking difference in collective cell morphology, where ECs lining the descending aorta are highly elongated and those lining the aortic arch have a cobblestone appearance (3). Endothelial cell alignment with flow induces a well-documented front-rear polarity with respect to the flow direction (4–6), including preferential polarization of the plasma membrane proteins NOTCH1 and vascular endothelial protein tyrosine phosphatase (VE-PTP) to the downstream end of ECs (7–9). ECs in the descending aorta also display a well-known antiinflammatory and atheroprotective phenotype (10), but the underlying mechanisms and connection to front-rear polarity are unknown. Further, the role of plasma membrane polarization for signaling compartmentalization and its link to antiinflammatory signaling in arterial ECs have not been described.

Plasma membrane compartmentalization has long been described as a means to achieve signaling specificity and efficiency. One mechanism for compartmentalization is through the formation of specialized plasma membrane domains, such as lipid rafts and/or caveolae, that can sequester signaling molecules (11, 12). Abundant in ECs, caveolae are known to play an important role in a variety of cellular functions, including signal transduction, endothelial nitric oxide synthase (eNOS) regulation, and calcium (Ca^2+^) signaling activity (13). In arterial ECs, localized Ca^2+^ “sparklets” via the ion channel transient receptor vanilloid 4 (TRPV4) have been shown to promote vasodilation by increasing intracellular Ca^2+^ (14, 15). TRPV4 is a cation-permeable ion channel whose activity is regulated by both direct and indirect mechanical activation (16). TRPV4 activity is known to regulate blood vessel homeostasis by enhancing vasodilation (17, 18). However, the role of endothelial TRPV4-mediated Ca^2+^ signaling in vascular inflammation is less clear (19–21).

Mechanotransduction is a critical contributor to EC resilience. Notably, laminar blood flow forces promote anti-oxidative, antithrombotic, and antiinflammatory effects that are atheroprotective and prevent EC dysfunction (22). In the face of stressors, cells alter their gene expression, protein synthesis, and signaling activities to restore homeostasis (23). Nevertheless, how flow inhibits inflammatory signaling to promote endothelial resilience and prevent EC dysfunction is incompletely understood (24). Endothelial dysfunction is characterized by a state of activation and proinflammatory signaling (25, 26). Although the inflammatory response can serve as a protective reaction to harmful stimuli, dysregulated vascular inflammation is a pathological driver of cardiovascular disease (27, 28).

Here, we investigated the role of sustained laminar flow in arterial EC signaling and found a distinct polarization of signaling activity, asymmetrically concentrated at the downstream end of cells. Polarization included membrane lipids, filamentous actin (F-actin), and caveolin-1 associated with TRPV4 channels. In the presence of high shear stress, localized Ca^2+^ oscillations via TRPV4 channels are sustained at these domains and contribute to eNOS activation and antiinflammatory gene expression. Our studies revealed a spatially restricted signaling domain in arterial ECs that is both mechanosensitive and antiinflammatory. The findings suggest that high laminar flow sustains a lateral polarity of ECs that is defined by flow direction through the asymmetric distribution of membrane components that regulate signaling activity. Furthermore, the data indicate that this signaling activity is an endothelial resilience mechanism and therefore offers a potential target for a therapeutic approach to reverse EC dysfunction and treat vascular inflammation.

## Results

### Front-rear polarization of arterial ECs.

**Figure 1:**
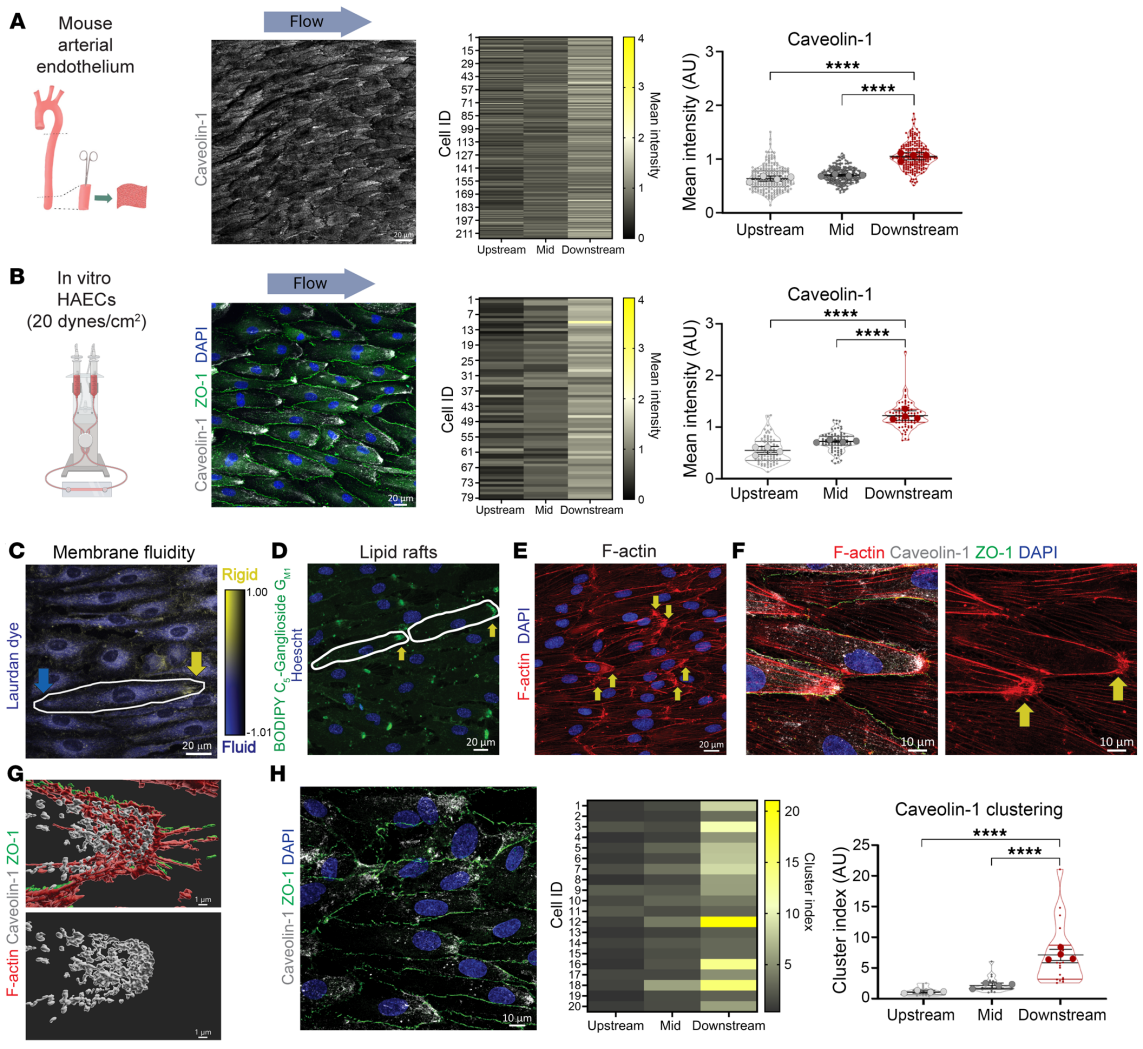
Membrane polarization and caveolin-1–enriched microdomains in aortic ECs exposed to laminar flow. (**A**) Confocal imaging of caveolin-1 in the endothelium of mouse descending aorta. Individual cells were segmented into 3 equal-length regions (upstream, mid-body, and downstream), and staining intensity was determined in each segment for 216 cells from *n* = 4 aortae. Graph displays mean ± SD with data analyzed by 1-way ANOVA and post hoc Tukey’s multiple-comparison test. Scale bar: 20 μm. (**B**–**H**) Confluent monolayers of HAECs were exposed to laminar shear stress (20 dynes/cm^2^) for 48 hours, then imaged. (**B**) HAECs were stained for caveolin-1, ZO-1, and DAPI, then segmented into 3 equal-length regions, and caveolin-1 staining intensity was quantified for each subcellular region for 79 cells from *n* = 4 biological replicates. Graph displays mean ± SD with data analyzed by 1-way ANOVA and post hoc Tukey’s multiple-comparison test. Scale bar: 20 μm. (**C**) Representative generalized polarization (GP) color-coded image to determine membrane fluidity. Higher GP was observed at the downstream end (yellow arrow) compared with the upstream end (blue arrow). Scale bar: 20 μm. (**D**) Staining of live cells with BODIPY FL C_5_-Ganglioside G_M1_ showed polarized accumulation of signal at the downstream end (arrows). Scale bar: 20 μm. (**E**) Fixed cells showed higher density of F-actin staining at downstream ends (arrows). Scale bar: 20 μm. (**F**) Imaging of F-actin, caveolin-1, ZO-1, and nuclei highlights presence of caveolin-1 and F-actin at the downstream end, and formation of F-actin web-like features (arrows). Scale bars: 10 μm. (**G**) 3D surface rendering of the downstream end of a cell showed high density of caveolin-1 where F-actin accumulates. Scale bars: 1 μm. (**H**) Representative image of caveolin-1, ZO-1, and nuclear staining used for caveolin-1 cluster analysis. Cluster index was determined in subcellular segments and plotted as means ± SD for *n* = 4 biological replicates. Data were analyzed by 1-way ANOVA and post hoc Tukey’s multiple-comparison test. Scale bar: 10 μm. ****P < 0.0001.

ECs lining the descending aorta are highly elongated, and we noted that high laminar flow resulted in a greater than 2-fold increase in EC aspect ratio in the descending aorta compared with the lower arch (Supplemental Figure 1A; supplemental material available online with this article; https://doi.org/10.1172/JCI175057DS1). To determine whether this elongated morphology was associated with altered caveolae organization, we stained ECs lining the mouse descending aorta for 2 components of caveolae, the plasma membrane–associated proteins caveolin-1 and cavin-1. We oriented the aorta based on the direction of blood flow, and quantified protein distribution by extracting fluorescence intensity in 3 cell segments of equal length, termed “upstream,” “mid-body,” and “downstream” in relation to the direction of flow (Supplemental Figure 1C). We found that the downstream end of descending aortic ECs displayed the highest levels of caveolin-1 and cavin-1 (Figure 1A and Supplemental Figure 1D).

To specifically investigate the connection between flow and protein distribution, we cultured human aortic EC (HAEC) monolayers on Y-shaped chambers and exposed them to unidirectional laminar flow for 48 hours. After 48 hours, monolayers of aligned cells displayed reduced migratory and proliferative behavior. HAECs in the high-flow region (~20 dynes/cm^2^) collectively aligned with the flow direction and were morphologically elongated, with a 3-fold increased cell aspect ratio compared with HAECs in the low-flow region (~5 dynes/cm^2^) (Supplemental Figure 1B). Importantly, the range of 5–20 dynes/cm^2^ represents the typical range of shear stress for large conduit arteries in humans (29). In line with in vivo observations, caveolin-1 and cavin-1 showed high concentration at the downstream end of HAECs exposed to high flow (Figure 1B and Supplemental Figure 1E).

We examined the broader cell surface asymmetries of flow-aligned HAECs by atomic force microscopy (Supplemental Figure 2A). This revealed that the cell’s downstream end was considerably stiffer (average Young’s modulus of 7.0 kPa) compared with the upstream end (average Young’s modulus of 5.3 kPa) (Supplemental Figure 2B). Furthermore, Laurdan dye imaging revealed asymmetry of the membrane fluidity across the ECs, where the downstream end typically exhibited a higher generalized polarization indicating higher membrane rigidity compared with the upstream end (Figure 1C). Membrane fluidity is a result of the distribution and composition of lipids within the bilayer. Accordingly, the fluorescent probe BODIPY FL C_5_-Ganglioside G_M1_ was strongly enriched at the downstream end of flow-aligned HAECs (Figure 1D), suggesting the presence of liquid-ordered or lipid raft domains in regions with enriched caveolin-1.

F-actin also displayed more densely webbed clusters at the downstream end of flow-aligned HAECs (Figure 1E). Importantly, these downstream F-actin clusters correlated with high-density caveolin-1 staining (Figure 1F). This was further highlighted for caveolin-1 and F-actin by 3D surface rendering (Figure 1G). Caveolin-2 was also observed to accumulate at the downstream end with caveolin-1 and F-actin clusters (Supplemental Figure 2, C–E). Markedly, the caveolin-1 cluster size was larger for HAECs exposed to high flow compared with low flow (Supplemental Figure 2F), and the cluster size increased about 6-fold within these polarized downstream areas in the presence of high flow (Figure 1H). In summary, exposure to sustained high laminar flow resulted in the formation of domains at the downstream end of HAECs, characterized by higher membrane rigidity, and the accumulation of raft-type lipids, F-actin aggregation, and caveolae-associated proteins caveolin-1, caveolin-2, and cavin-1.

### Activation of eNOS and Ca^2+^ entry occur at the downstream end.

**Figure 2:**
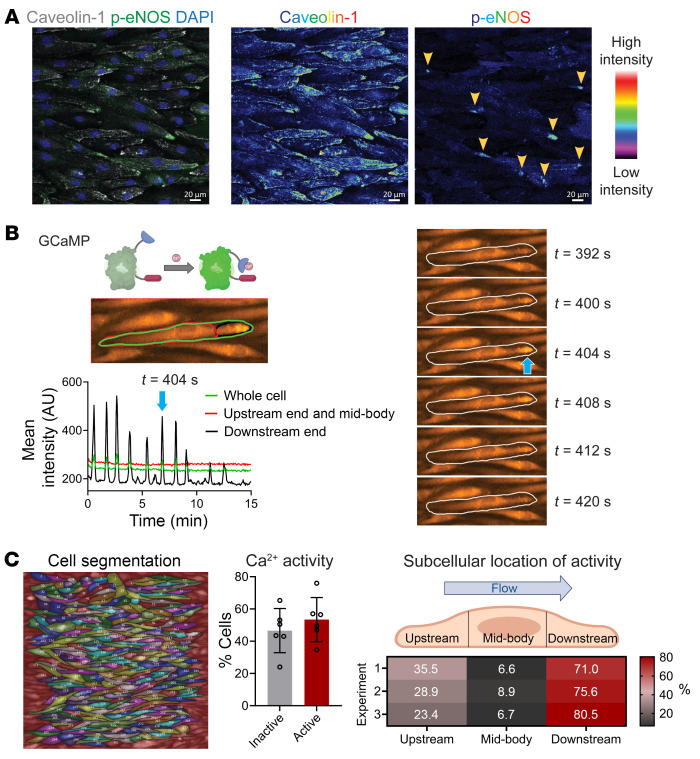
eNOS phosphorylation and Ca^2+^ oscillations occur at the downstream end in the presence of high laminar flow. (**A**–**C**) Confluent monolayers of HAECs were exposed to laminar shear stress (20 dynes/cm^2^) for 48 hours. (**A**) Representative image of flow-aligned HAECs stained for caveolin-1 and eNOS phosphorylated on serine 1177 (p-eNOS). Individual channel images, displayed in rainbow lookup table, highlight the accumulation of signal for both caveolin-1 and p-eNOS at the downstream end (arrowheads). Scale bars: 20 μm. (**B**) Imaging of Ca^2+^ activity in live HAECs expressing GCaMP. Fluorescence intensity over time is plotted for 15 minutes for 1 full-length cell using 3 defined regions of interest. Note that Ca^2+^ oscillations are observed exclusively at the downstream end; blue arrow indicates one Ca^2+^ peak. Corresponding time sequence is displayed for indicated time points. (**C**) All cells within an imaging field of view were outlined and assigned ID numbers. GCaMP signal was extracted over 30 minutes for *n* = 6 independent experiments and analyzed for Ca^2+^ activity. Approximately 50% of cells had Ca^2+^ activity (index of dispersion [IoD] greater than 2). From 730 cells (*n* = 3 independent experiments), active cells were further segmented into 3 equal-length segments for the upstream, mid-body, and downstream regions. Of the active cells, over 70% had Ca^2+^ activity restricted to the downstream end.

Given the known functional connections between caveolae and eNOS (30–32), we hypothesized that polarization of caveolin-1–rich membrane domains led to localized eNOS activation. Indeed, eNOS phosphorylated on serine 1177 (p-eNOS), an active form of eNOS, was found predominantly concentrated at the downstream end of flow-aligned HAECs, with its staining mostly overlapping with that of the polarized caveolin-1 clusters (Figure 2A). Thus, caveolin-1 polarization correlates with localized activation of eNOS at the downstream end of ECs exposed to sustained flow.

Phosphorylation of eNOS on serine 1177 is frequently Ca^2+^ dependent (33, 34). To examine changes in intracellular free Ca^2+^, we transfected HAECs with plasmids encoding the Ca^2+^ reporter GCaMP and exposed them to high laminar flow (Figure 2B and Supplemental Video 1). Segmentation analysis of individual cells revealed that oscillatory Ca^2+^ influx events were restricted to the downstream end (black) and whole cell (green), which we identified as enriched for caveolin-1 and p-eNOS (Figure 2B and Supplemental Videos 2 and 3). To quantify the prevalence of Ca^2+^ activity across the monolayer, full-length cells were segmented, and “active” cells were identified as having an index of dispersion greater than 2. Approximately 50% of the cells showed Ca^2+^ transients over a 30-minute imaging period (Figure 2C), indicating that oscillatory Ca^2+^ influx events were a sustained response of ECs under high laminar flow. Segmentation analysis of these active cells revealed that transients were restricted to the downstream end in over 70% of the active cells (Figure 2C and Supplemental Figure 3A).

### Polarized signaling activity requires high laminar flow.

**Figure 3:**
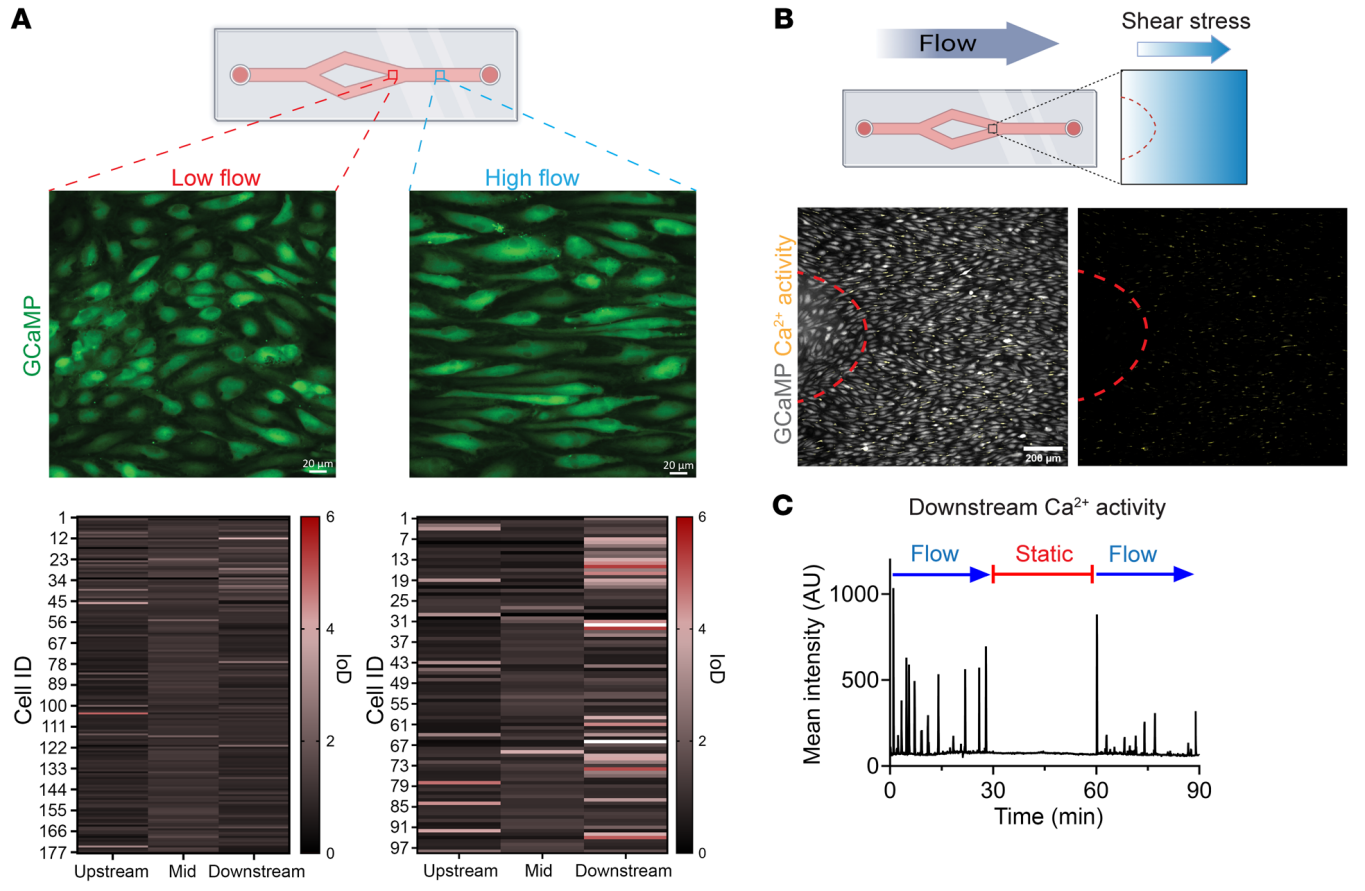
Ca^2+^ activity at the downstream end is enhanced with increased shear stress. (**A**) To model variation in flow, GCaMP HAECs were seeded on Y-shaped slides and exposed to unidirectional laminar flow for 48 hours before imaging. Ca^2+^ activity was imaged in low-flow (~5 dynes/cm^2^) and high-flow regions (~20 dynes/cm^2^). Cell segmentation and extraction of the fluorescence intensity over time showed enhanced activity at the downstream end of cells in the high-flow region. IoD plots show data from 177 low-flow and 98 high-flow cells across *n* = 3 biological replicates. Scale bars: 20 μm. (**B**) Region of flow convergence on the Y-shaped slide experiences increasing shear stress levels. Cells exposed to high flow were morphologically aligned and exhibited more Ca^2+^ spikes (in yellow) compared with cells in the low-flow area (marked by red outline). Scale bar: 200 μm. (**C**) Representative GCaMP intensity trace showing 90 minutes of Ca^2+^ activity at the downstream end of flow-aligned HAECs in the presence of flow (30 minutes; 20 dynes/cm^2^), static (30 minutes; 0 dynes/cm^2^), and re-flow (30 minutes; 20 dynes/cm^2^) conditions.

We next compared Ca^2+^ activity for HAECs in low-flow versus high-flow regions on Y-slides and observed that elevated shear stress correlated with increased Ca^2+^ oscillations. In the low-flow region, cells were less elongated and exhibited fewer Ca^2+^ oscillations, which were not preferentially localized (Figure 3A and Supplemental Videos 4 and 5). With increasing shear stress, cell morphology changed from cobblestone to elongated, and we observed concomitant enhanced Ca^2+^ activity with increasingly preferential localization to the downstream end of the elongated cells (Figure 3B, Supplemental Figure 3B, and Supplemental Video 6). In fact, there was a positive correlation between cell aspect ratio and Ca^2+^ oscillations at the downstream end (Supplemental Figure 3C), suggesting that flow-induced elongation is required for the localized Ca^2+^ signaling.

To capture the process of EC alignment with flow and Ca^2+^ activity, we imaged GCaMP-HAECs over the 48 hours, recording for 10 minutes every hour. We observed that Ca^2+^ activity at the downstream end of the cell was sustained only at the conclusion of flow alignment when cells had established an increased aspect ratio (Supplemental Figure 3D and Supplemental Video 7). Tracking an individual cell over the 48 hours, we noted that the prevalence and location of Ca^2+^ activity were sporadic as the cell changed shape, with localized activity when the cell aspect ratio was greater than 4 (Supplemental Figure 3E and Supplemental Video 8), further indicating that EC alignment with flow sustains the localized Ca^2+^ oscillations at the downstream end. To test whether non-arterial ECs exhibit this signaling behavior in response to flow alignment, we imaged GCaMP-transfected human umbilical vein ECs (HUVECs) under arterial flow (20 dynes/cm^2^) and observed sustained Ca^2+^ oscillations at the downstream end of elongated cells (Supplemental Figure 4A and Supplemental Video 9). The flow-aligned HUVEC monolayer also displayed a polarized accumulation of caveolin-1 at the downstream end (Supplemental Figure 4, B and C), suggesting a response similar to that seen in arterial ECs. To test the requirement of shear stress for the Ca^2+^ signaling, we imaged GCaMP-HAECs over the time course of flow, static, and re-flow conditions, which revealed that the oscillatory Ca^2+^ activity was observed only in the presence of laminar flow (Figure 3C, Supplemental Figure 4D, and Supplemental Video 10).

**Figure 4:**
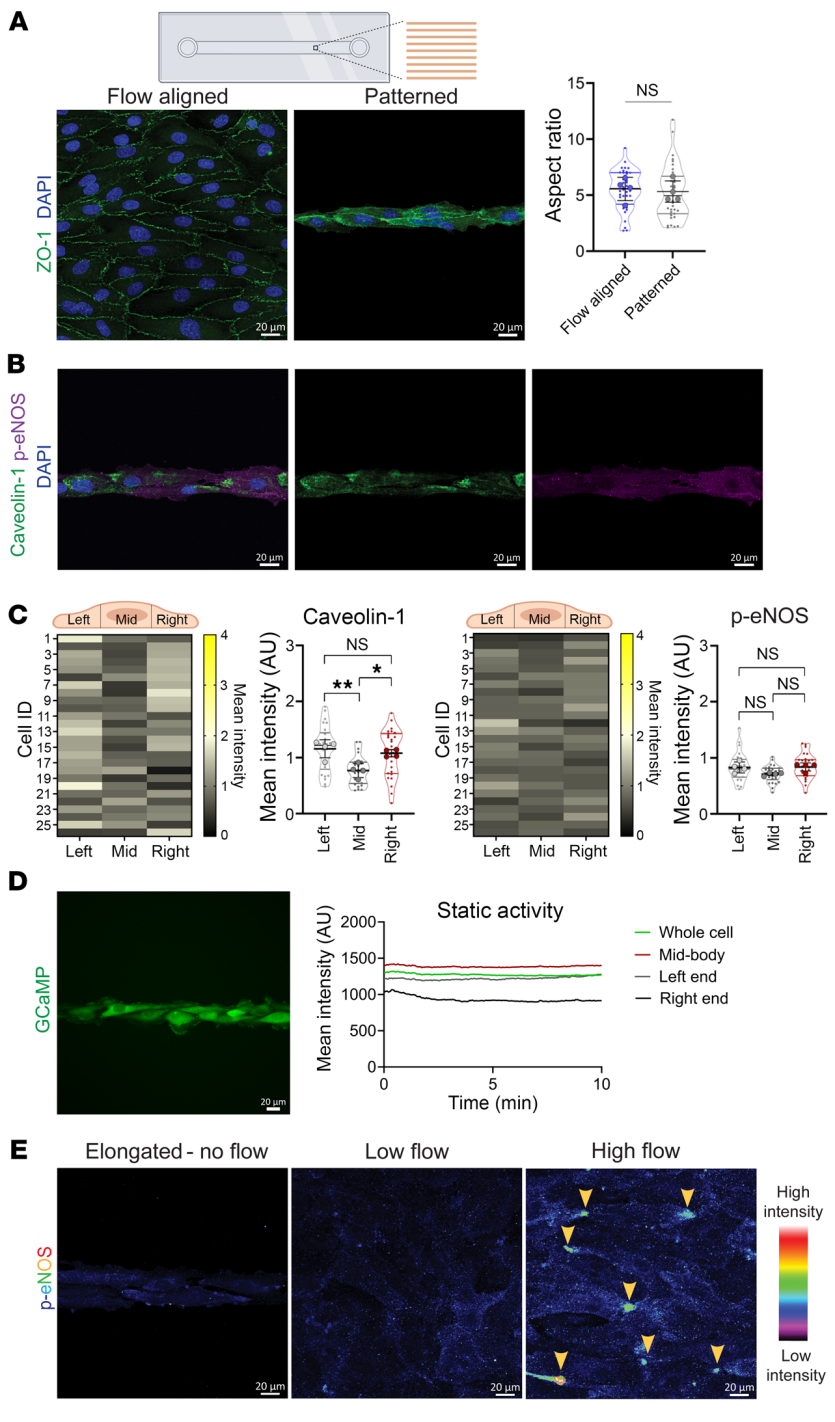
High laminar flow is required for localized signaling activity. (**A**) HAECs were either seeded on non-patterned chambers and flow-aligned (20 dynes/cm^2^) or seeded on line-patterned chambers and cultured statically. After 48 hours, cells were analyzed and aspect ratio calculated for *n* = 4 biological replicates. Shown are means ± SD; NS, not significant by 2-tailed, unpaired *t* test. Scale bars: 20 μm. (**B** and **C**) HAECs were elongated statically on the line-patterned chamber and stained for caveolin-1, p-eNOS, and DAPI. Representative images of the staining are shown in **B**, with segmentation analysis from 26 cells shown in **C**. Graphs show intensity displayed as means ± SD and analyzed by 1-way ANOVA with post hoc Tukey’s multiple-comparison test. *P < 0.05, **P < 0.01. Scale bars: 20 μm. (**D**) Ca^2+^ activity was recorded for GCaMP-expressing HAECs that were cultured statically on line-patterned chambers. Representative live cell recording of intensity trace for 1 cell over 10 minutes showed a lack of localized activity. Scale bar: 20 μm. (**E**) HAECs were cultured statically on the line-patterned chamber or Y-shaped slide for 48 hours. Representative images of p-eNOS staining using equivalent imaging conditions to compare the signal intensity across conditions. The p-eNOS signal is displayed using false-color rainbow lookup table to highlight the clustered regions of staining in cells under high flow. Scale bars: 20 μm.

We next asked whether cell body elongation is sufficient for these the flow-induced phenotypes. To test this, we elongated HAECs in the absence of flow, by culturing them on line-patterned slides to achieve cell aspect ratio similar to those seen with flow (Figure 4A). Indeed, caveolin-1 showed asymmetric distribution at one end of static, elongated cells (Figure 4B). However, in contrast with flow-aligned HAECs, static elongated ECs accumulated caveolin-1 on either end of the cell, resulting in a random pattern. Importantly, polarized p-eNOS was not observed in static elongated ECs (Figure 4C). Accordingly, they did not display Ca^2+^ oscillations as measured by live cell imaging of GCaMP-HAECs (Figure 4D and Supplemental Video 11). Unlike HAECs exposed to high flow, HAECs exposed to low flow or static elongated HAECs did not show asymmetric activation of p-eNOS (Figure 4E). Thus, while cell elongation was sufficient to trigger caveolin-1 clustering at either end of ECs, the sustained presence of high laminar flow was required for coordinated polarization of caveolin-1 within the population and the formation of functional signaling domains.

### Localized Ca^2+^ entry occurs via TRPV4/caveolin-1 association at the downstream end.

**Figure 5:**
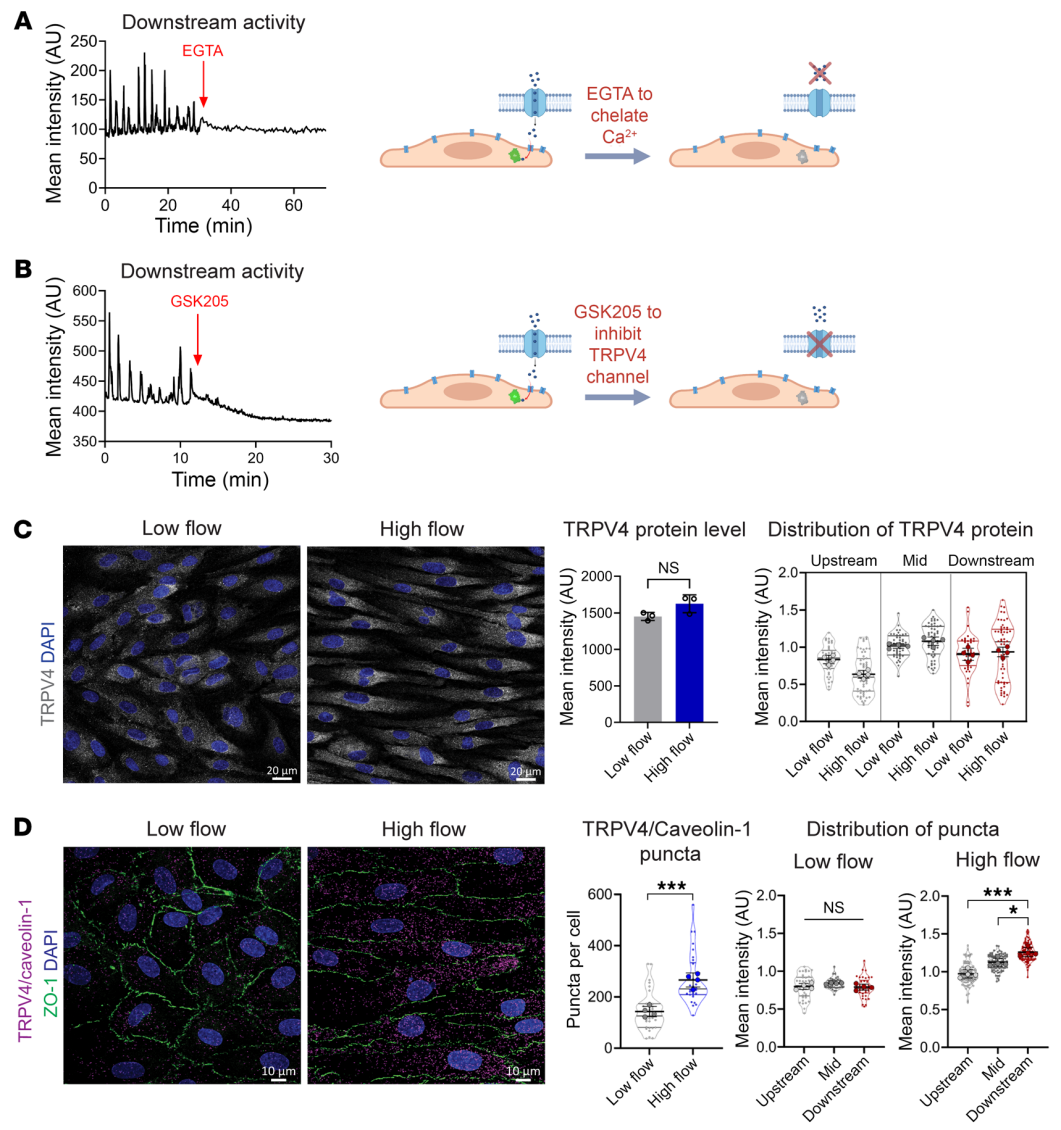
Localized Ca^2+^ entry requires TRPV4 channel activity and occurs in areas of TRPV4/caveolin-1 association. (**A** and **B**) Confluent monolayers of GCaMP-transfected HAECs were exposed to laminar shear stress (20 dynes/cm^2^) for 48 hours before live cell imaging. (**A**) Representative GCaMP intensity trace showing Ca^2+^ activity at the downstream end after the addition of EGTA (1.6 μM) to chelate calcium ions in culture medium. (**B**) Representative GCaMP intensity trace showing Ca^2+^ activity at the downstream end after the addition of the TRPV4 antagonist GSK205 (20 μM). (**C** and **D**) HAECs were seeded on Y-shaped slides and exposed to unidirectional laminar flow for 48 hours. Immunofluorescence was compared for cells in low-flow (~5 dynes/cm^2^) and high-flow regions (~20 dynes/cm^2^). (**C**) TRPV4 protein staining showed no difference for low-flow versus high-flow regions. Representative images from *n* = 3 biological replicates; statistics calculated by 2-tailed, unpaired *t* test show no significance (NS) of difference between regions. Quantifying the subcellular distribution of expression indicated that TRPV4 was not polarized under flow. Shown are means ± SD from 44 low-flow and 57 high-flow cells across 4 biological replicates. Scale bars: 20 μm. (**D**) Representative images of proximity ligation assay (PLA) to detect TRPV4 and caveolin-1 association (magenta puncta) in low-flow and high-flow regions for *n* = 4 replicates. Shown are puncta per cell with means ± SD and statistics calculated using unpaired, 2-tailed *t* test. ***P < 0.001. Additional segmentation analysis showed that TRPV4/caveolin-1 PLA puncta preferentially occurred in the downstream end only for cells exposed to high flow. Thirty-eight cells were analyzed for the low-flow region and 93 cells for the high-flow region from *n* = 4 biological replicates. Data were analyzed by 1-way ANOVA and post hoc Tukey’s multiple-comparison test. *P < 0.05, ***P < 0.001. Scale bars: 10 μm.

We postulated that Ca^2+^ entry from the extracellular space via plasma membrane channels was required for Ca^2+^ activity. Consistent with this, the addition of the Ca^2+^ chelator EGTA blunted these Ca^2+^ oscillations (Figure 5A and Supplemental Figure 5A). Furthermore, Ca^2+^ oscillations at the downstream end continued in the presence of cyclopiazonic acid (CPA) (Supplemental Figure 5B), a sarco/endoplasmic reticulum Ca^2+^-ATPase inhibitor, suggesting that the observed activity was independent of Ca^2+^ release from intracellular stores. Since the ion channel TRPV4 is implicated in endothelial Ca^2+^ “sparklet” activity in mouse arteries (14), we tested the role of TRPV4 channels by adding the TRPV4-specific antagonist GSK205 during imaging of GCaMP-HAECs under flow and found that it suppressed Ca^2+^ activity (Figure 5B and Supplemental Figure 5C). Considering the role of PIEZO channels as sensors of blood flow (35, 36), we also tested the role of PIEZO1 by adding the stretch-activated cation channel blocker GsMTx-4 (37), which did not affect Ca^2+^ oscillations at the downstream end (Supplemental Figure 5D). The cumulative data indicate that TRPV4 is required for oscillatory Ca^2+^ entry at polarized caveolin-1–rich domains, where active eNOS was observed in flow-exposed elongated ECs.

Next, we investigated how TRPV4-dependent Ca^2+^ entry was polarized in response to flow conditioning of HAECs. Notably, TRPV4 mRNA and protein levels were indistinguishable in HAECs irrespective of the presence of laminar flow, whereas known flow-responsive genes *KLF2* and *KLF4* and eNOS protein showed an upregulation in response to flow (Supplemental Figure 5, E and F). Importantly, unlike caveolin-1, caveolin-2, or cavin-1, TRPV4 was uniformly distributed across the plasma membrane and did not display any apparent polarization (Figure 5C).

TRPV4 interacting with caveolin-1 leads to its activation in lung endothelium (38). To determine whether this association also occurred in flow-conditioned HAECs, we used a proximity ligation assay (PLA). We observed specific in situ PLA spots when combining antibodies against caveolin-1 and TRPV4 or cavin-1, but not histone H3, and not with individual antibodies alone (Supplemental Figure 5G). TRPV4/caveolin-1 PLA spots were strongly enhanced in HAECs exposed to high flow and also clearly accumulated at the downstream end of the elongated cells. Cells in the low-flow region did not show a difference in the distribution of PLA spots (Figure 5D). Combined, these data indicated that TRPV4 associated with caveolin-1 clusters in the presence of high flow at the downstream end of cells where oscillatory TRPV4-mediated Ca^2+^ activity occurs.

### Disruption of polarized caveolin-1 abolishes localized Ca^2+^ activity.

**Figure 6:**
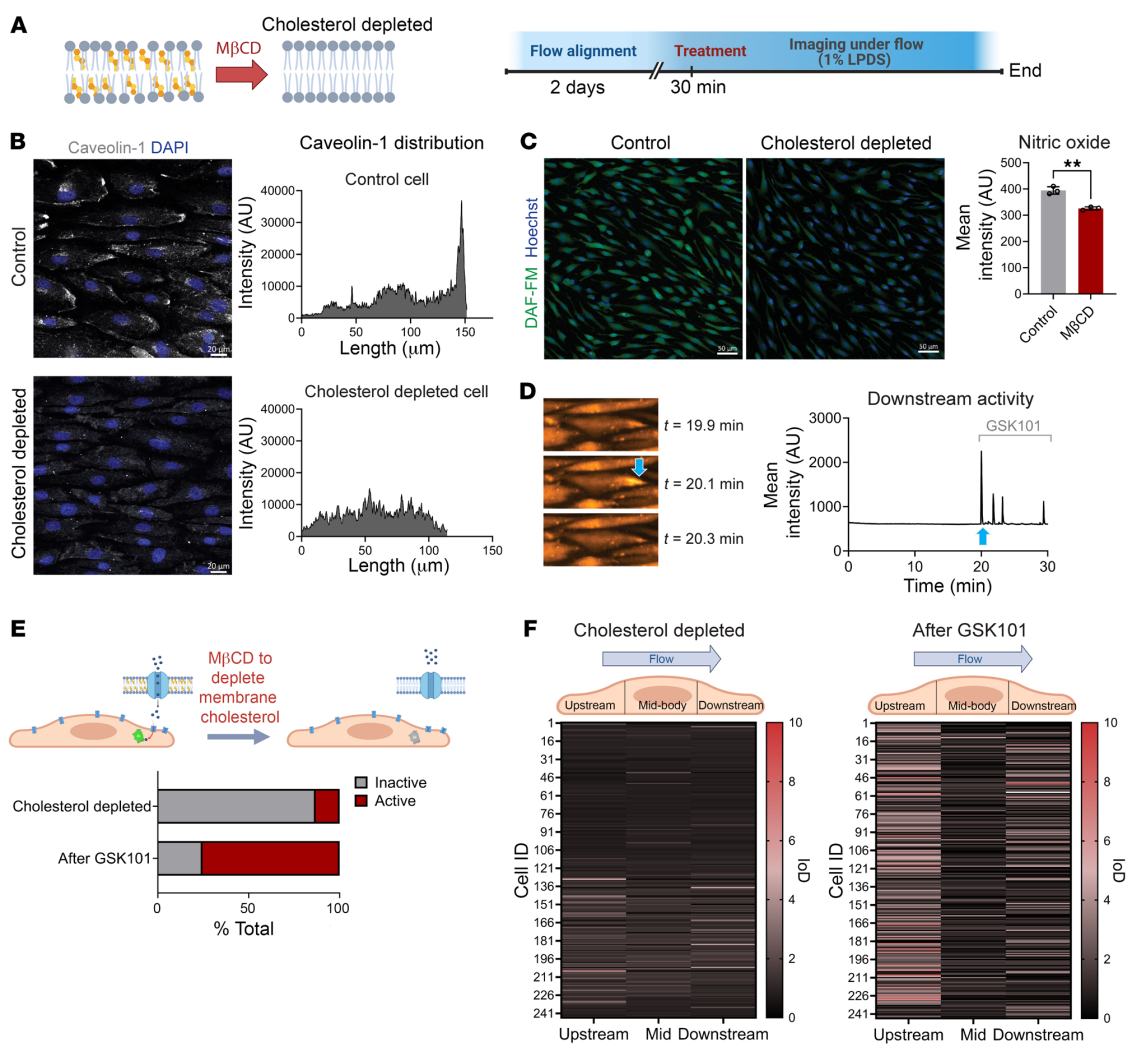
Cholesterol depletion abolishes polarized signaling. (**A**) Experimental design for cholesterol depletion of HAECs. MβCD was used to deplete plasma membrane cholesterol. (**B**) Flow-aligned HAECs were treated with MβCD for 30 minutes, then fixed and stained for caveolin-1 and DAPI. MβCD treatment abolished caveolin-1 polarization as shown by intensity plots of representative cells from the control and MβCD-treated groups. Scale bars: 20 μm. (**C**) NO production was visualized via DAF-FM–loaded flow-aligned monolayers of control and MβCD-treated HAECs. Shown are mean DAF-FM fluorescence intensities ± SD for *n* = 3 biological replicates and statistics calculated using 2-tailed, unpaired *t* test. **P < 0.01. Scale bars: 50 μm. (**D**) GCaMP imaging of the cholesterol-depleted cells under flow (20 dynes/cm^2^) for 20 minutes showed lack of Ca^2+^ activity. Displayed are time-dependent images of a representative cell and the corresponding intensity trace for the downstream end. At *t* = 20 minutes (blue arrow), the TRPV4 agonist GSK1016709A (GSK101; 10 nM) was added to the flowing culture medium. This led to an immediate Ca^2+^ burst as seen in the image at 20.1 minutes. (**E**) Overall, only 13% of the cells depleted for cholesterol were active in the initial 20 minutes of imaging. The number of active cells increased to 75% following the addition of GSK101. (**F**) IoD heatmaps show Ca^2+^ activity following cholesterol depletion and subsequent GSK101 addition for *n* = 244 cells.

Cholesterol affects caveolin-1 clustering and functions of caveolae, and sequestration of cholesterol using methyl-β-cyclodextrin (MβCD) disrupts caveolae function (39). We treated flow-aligned HAECs with MβCD for 30 minutes (Figure 6A). This effectively depleted the accessible pool of cholesterol (Supplemental Figure 6A) and also disrupted flow-induced plasma membrane polarization of caveolin-1 (Figure 6B). Functionally, this treatment reduced the levels of intracellular nitric oxide (NO) (Figure 6C), further supporting that caveolin-1 polarization directly affects endothelial signaling. Indeed, this also resulted in a loss of localized Ca^2+^ activity under laminar flow (Figure 6, D–F). Furthermore, treatment with the TRPV4-specific agonist GSK1016790A (GSK101; 10 nM) led to Ca^2+^ activity at the ends of elongated ECs exposed to MβCD (Figure 6, D–F), indicating that TRPV4 channels continued to be activatable in the absence of cholesterol. In line with our observation that TRPV4 channel expression was observed across the entire cell surface (Figure 5C), we found that GSK101 stimulated Ca^2+^ entry across the entire plasma membrane (Supplemental Figure 6B). However, higher doses (1 μM) were required to induce Ca^2+^ entry throughout the entire cell body, suggesting that the TRPV4 channel open probability was greater at the ends of flow-elongated ECs.

### Inhibition of TRPV4 activity in the presence of laminar flow results in inflammation.

**Figure 7:**
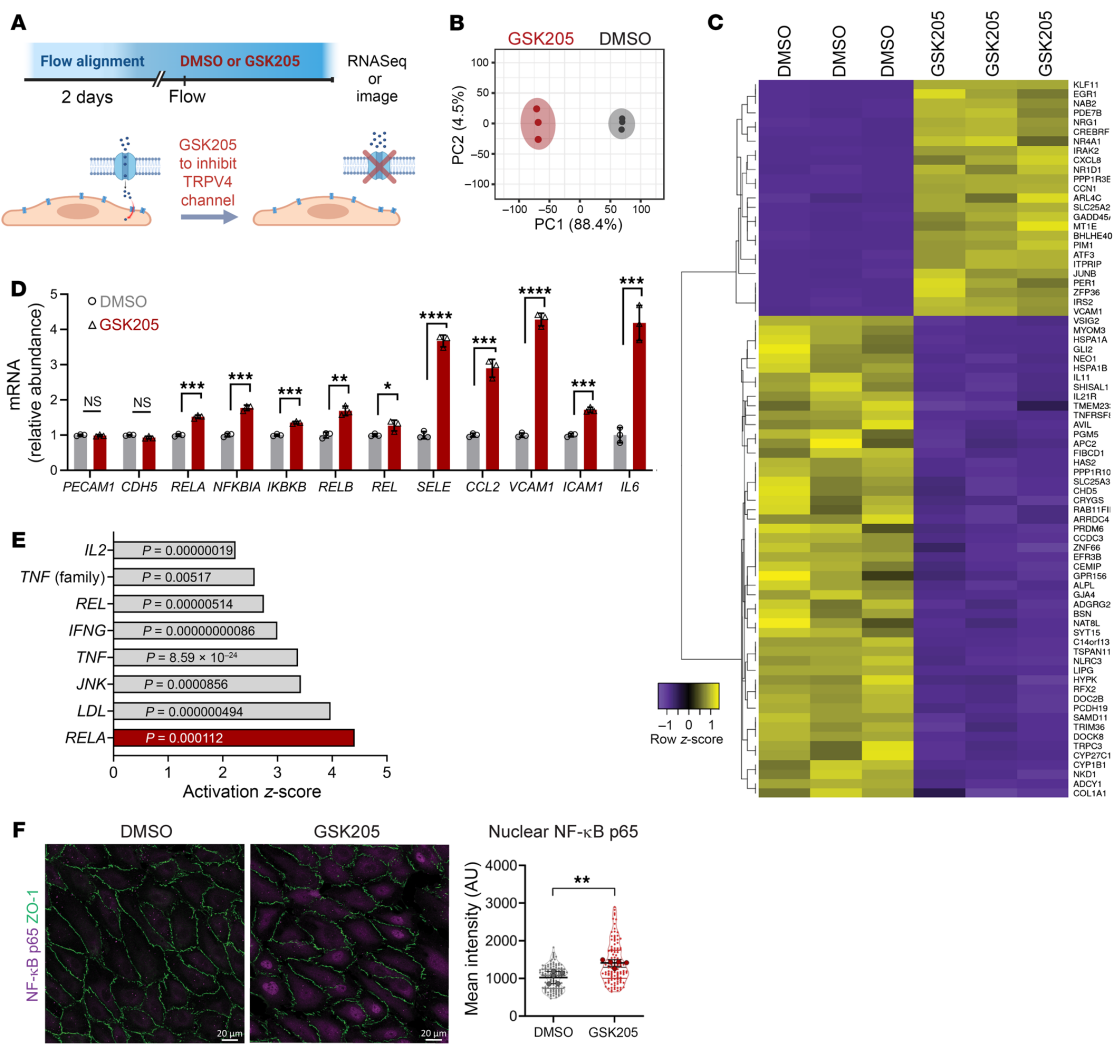
Inhibition of TRPV4 activity under flow induces inflammatory gene expression and NF-κB signaling. (**A**) HAEC monolayers were flow-aligned for 48 hours followed by the addition of DMSO (control) or TRPV4 antagonist GSK205 (20 μM) in the presence of laminar flow (20 dynes/cm^2^). (**B**) Principal component analysis (PCA) plot of samples based on RNA sequencing analysis. RNA from *n* = 3 replicates per condition. (**C**) Heatmap displays top 76 significantly expressed genes between GSK205- and DMSO-treated HAECs. (**D**) Bar plot of gene expression shows an increase in inflammatory gene expression for GSK205-treated HAECs. Statistics calculated using 2-tailed, unpaired *t* test. *P < 0.05, **P < 0.01, ***P < 0.001, ***P < 0.0001. (**E**) Bar plot of the top putative upstream regulators in response to GSK205 treatment as identified by Ingenuity Pathway Analysis (IPA) of the RNA sequencing data. (**F**) Control and GSK205-treated monolayers were fixed after 2 hours and stained for ZO-1 and the p65 subunit of NF-κB. Nuclear expression of NF-κB p65 was quantified by mean fluorescence intensity. Shown is mean intensity from *n* = 117 (DMSO) and *n* = 115 (GSK205) cells analyzed from *n* = 5 biological replicates and statistics calculated using 2-tailed, unpaired *t* test. **P < 0.01. Scale bars: 20 μm.

We next investigated whether flow-induced TRPV4 activity promotes an antiinflammatory signaling behavior. For this, we modulated TRPV4 activity in flow-aligned HAECs by treating them with the TRPV4 antagonist GSK205 or vehicle, and performed transcriptional profiling and confocal imaging (Figure 7A). Inhibition of TRPV4 in flow-aligned HAECs resulted in an altered transcriptional profile after 2 hours (Figure 7, B and C, and Supplemental Figure 7A). Specifically, we noted the emergence of an inflammatory phenotype, as evidenced by Kyoto Encyclopedia of Genes and Genomes (KEGG) pathway analysis and gene set enrichment analysis of the RNA sequencing data (Supplemental Figure 7, B and C). Individual gene expression changes by quantitative PCR (qPCR) confirmed an upregulation of inflammatory genes associated with NF-κB activation, including *SELE*, *VCAM1*, and *ICAM1* (Figure 7D). Ingenuity Upstream Regulator Analysis in IPA (QIAGEN) of the RNA sequencing data revealed the NF-κB gene *RELA*, which is the NF-κB p65 subunit, to be the top activated upstream regulator in response to GSK205 treatment (Figure 7E).

**Figure 8:**
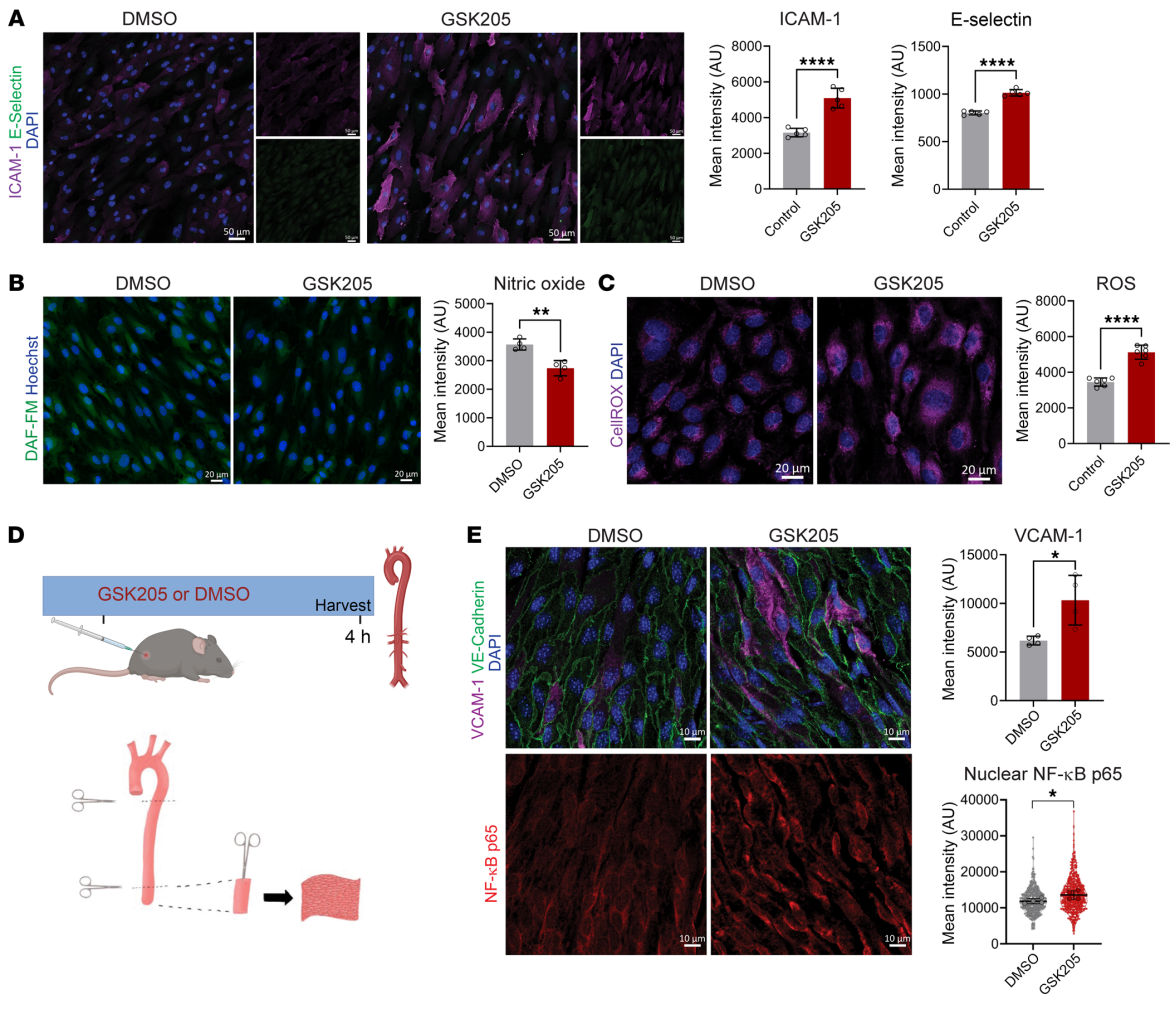
Inhibition of TRPV4 activity induces an inflammatory phenotype. (**A**) Control and GSK205-treated HAECs were fixed after 4 hours and stained for DAPI, ICAM-1, and E-selectin. Shown are representative images and mean intensities ± SD from *n* = 5 biological replicates and statistics calculated using 2-tailed, unpaired *t* test. ****P < 0.0001. Scale bars: 50 μm. (**B**) Quantification of NO production in live cells by DAF-FM imaging after 2 hours of treatment. Shown are representative images and mean intensities ± SD from *n* = 4 biological replicates and statistics calculated using 2-tailed, unpaired *t* test. **P = 0.0023. Scale bars: 20 μm. (**C**) After 2 hours, control and GSK205-treated monolayers were incubated with CellROX probe and imaged to quantify ROS production. Shown are representative images and mean intensities ± SD from *n* = 6 biological replicates and statistics calculated using 2-tailed, unpaired *t* test. ****P < 0.0001. Scale bars: 20 μm. (**D**) Experimental design for in vivo inhibition of TRPV4 activity via i.p. injection of GSK205 (10 mg/kg) or DMSO (volume equivalent) as control. After 4 hours, mice were euthanized and aortae collected for en face imaging. (**E**) Confocal imaging of abdominal aortae from mice injected with GSK205 or DMSO. Shown are representative images and associated quantification of VCAM-1 and nuclear NF-κB p65 staining intensity. Graphs represent mean intensities ± SD from *n* = 4 mice and statistics calculated using 2-tailed, unpaired *t* test. *P < 0.05. Scale bars: 10 μm.

In line with the changes in gene expression, 2-hour GSK205 treatment was associated with enhanced nuclear localization of the NF-κB p65 subunit (Figure 7F). By contrast, treatment with GsMTx-4 for 2 hours did not result in nuclear NF-κB p65 (Supplemental Figure 8A), further indicating that the inflammatory response was not associated with PIEZO channels. Moreover, inhibition of TRPV4 activity for 4 hours increased surface expression of the proinflammatory adhesion molecules ICAM-1 and E-selectin (Figure 8A). Inhibition of TRPV4 activity also resulted in reduced NO production (Figure 8B) and increased reactive oxygen species (ROS) generation (Figure 8C), whereas GSK101-mediated activation of TRPV4 enhanced NO production (Supplemental Figure 8B). We confirmed that caveolin-1–rich domains remained polarized during TRPV4 inhibition (Supplemental Figure 8C), suggesting that the domains are present but not signaling. Finally, siRNA-mediated reduction of TRPV4 protein in HAECs exposed to laminar flow also reduced eNOS levels (Supplemental Figure 8D) and increased nuclear NF-κB p65 (Supplemental Figure 8E). We infer that TRPV4 activity in ECs exposed to laminar flow prevents proinflammatory responses in vitro.

To test the role of TRPV4 activity in vivo, we injected wild-type mice with either GSK205 (10 mg/kg) or vehicle and analyzed the aorta 4 hours later (Figure 8D). En face staining of the aortic endothelium showed increased expression of VCAM-1 and nuclear NF-κB p65 in mice treated with GSK205 (Figure 8E). Taken together, these data indicated that TRPV4 activity sustains a pro-vasodilatory and antiinflammatory phenotype in the presence of laminar flow in vitro and in the aorta in vivo.

### Activation of TRPV4 ameliorates the response to an acute inflammatory stimulus.

**Figure 9:**
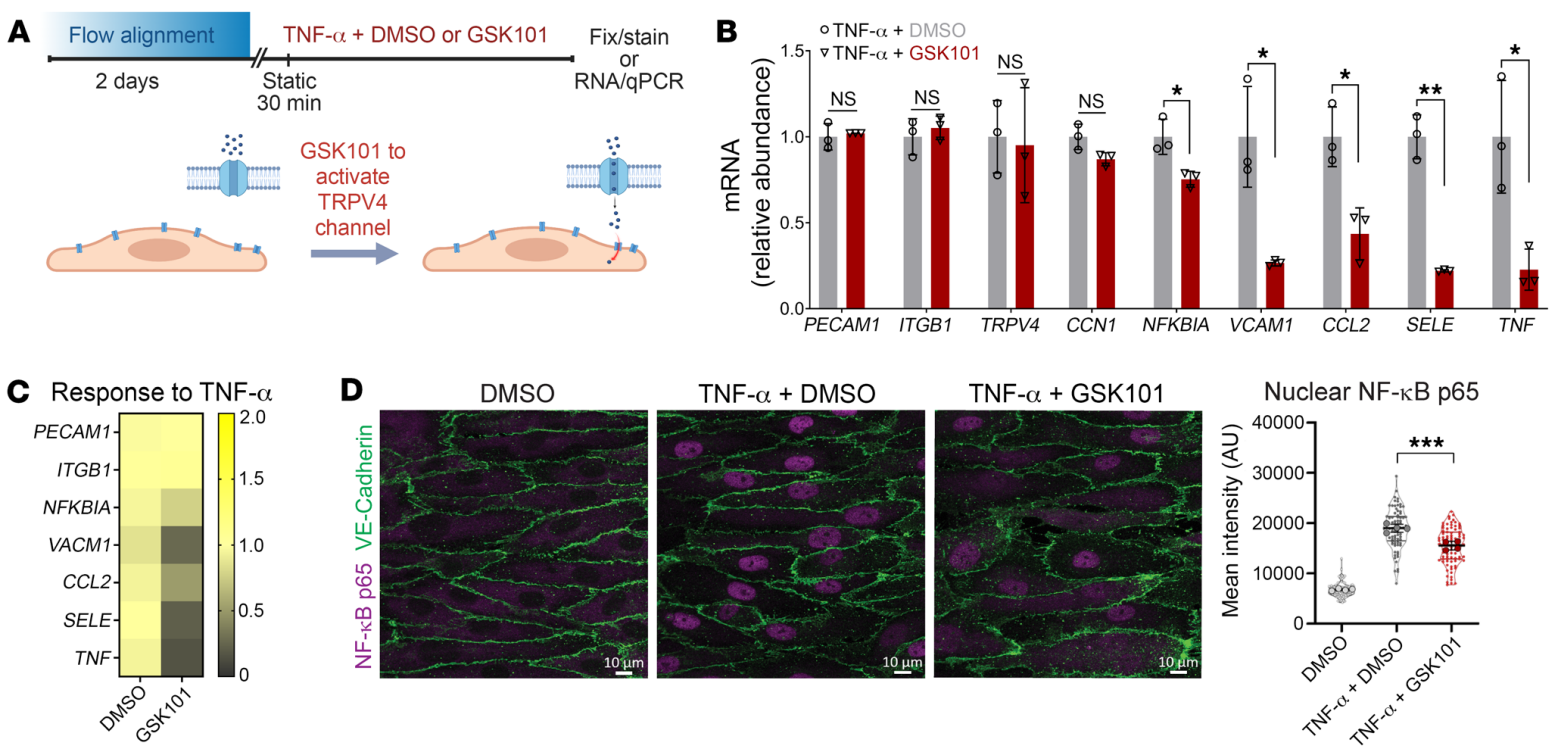
Activation of TRPV4 dampens the endothelial response to an inflammatory stimulus in vitro. (**A**) Experimental design for static TNF-α treatment (10 ng/mL; 30 minutes) in the presence of GSK101 (10 nM) or DMSO. (**B**) Gene expression was measured by qPCR for TNF-α–treated monolayers in the presence of DMSO or GSK101. Gene expression of *PECAM1*, *ITGB1*, *TRPV4*, *CCN1*, *NFKBIA*, *VCAM1*, *CCL2*, *SELE*, and *TNF* plotted as mean ± SD. *P < 0.05, **P < 0.01 by 2-tailed, unpaired *t* test; *n* = 3 biological replicates. (**C**) Gene expression as in **B** with mRNA expression plotted as a heatmap of mean expression. (**D**) After TNF-α treatment with or without GSK101, HAECs were fixed and stained for VE-cadherin and NF-κB p65. Graph represents mean fluorescence intensity in the nucleus from *n* = 110 (DMSO), *n* = 79 (TNF-α + DMSO), and *n* = 87 (TNF-α + GSK101) cells per group from *n* = 4 biological replicates per condition and statistics calculated using 1-way ANOVA with post hoc Tukey’s multiple-comparison test. ***P < 0.001. Scale bars: 10 μm.

We considered that activation of TRPV4 could enhance cell resilience and an antiinflammatory response in ECs. Caveolin-1–rich domains remained polarized for at least 30 minutes after removal of flow (Supplemental Figure 9A), so we used flow-aligned HAECs in static conditions to study the effect of activating TRPV4 in the presence of tumor necrosis factor-α (TNF-α) (Figure 9A). Treatment with GSK101 (10 nM) for 30 minutes reduced the expression of proinflammatory genes, including NF-κB inhibitor α (*NFKBIA*), vascular cell adhesion molecule 1 (*VCAM1*), C-C motif chemokine ligand 2 (*CCL2*), selectin E (*SELE*), and *TNF* (Figure 9, B and C). Nuclear localization of NF-κB p65 was also attenuated upon activation of TRPV4 (Figure 9D). Changes to ICAM-1 protein expression levels were not observed within this short inflammatory TNF-α stimulation (Supplemental Figure 9B). However, we observed TNF-α–stimulated ROS production, which was attenuated by GSK101 costimulation (Supplemental Figure 9C). In contrast, the inflammatory response to TNF-α was not reduced by activation of PIEZO1 channels with the agonist Yoda1 (Supplemental Figure 10, A–C), further supporting the role of TRPV4 channels in mitigating inflammation. Overall, these data indicate that exogenous TRPV4 activation suppresses the inflammatory TNF-α response in HAECs.

**Figure 10:**
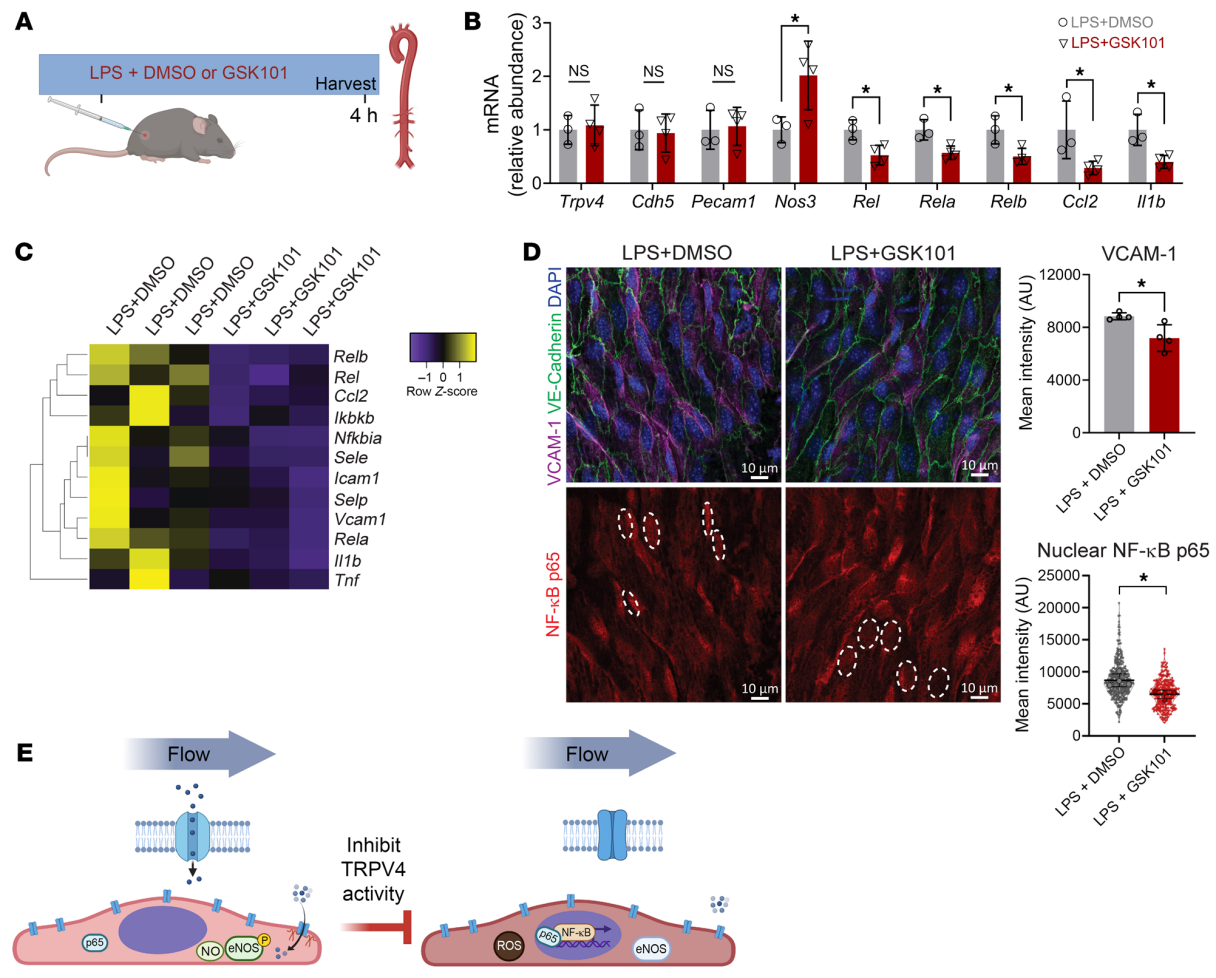
Activation of TRPV4 attenuates endothelial response to an inflammatory stimulus in vivo. (**A**) Experimental design for in vivo LPS treatment (1.5 mg/kg) in the presence of GSK101 (10 μg/kg) or DMSO in wild-type mice. (**B**) Gene expression of aortic endothelium quantified by qPCR of mRNA isolated from the descending aorta of mice injected with LPS plus DMSO (*n* = 3) or LPS plus GSK101 (*n* = 4). Gene expression plotted as mean ± SD; *P < 0.05 by 2-tailed, unpaired *t* test. (**C**) Aortic endothelial gene expression plotted as heatmap for inflammatory genes measured by qPCR in **B**. (**D**) En face staining of abdominal aortae from mice injected with LPS plus DMSO or LPS plus GSK101. Shown are representative images and associated quantification of VCAM-1 staining intensity and NF-κB p65 nuclear intensity. Graphs represent mean intensities ± SD from *n* = 4 mice and statistics calculated using 2-tailed, unpaired *t* test. *P < 0.05. Scale bars: 10 μm. (**E**) Graphical model describing how laminar flow supports localized TRPV4 activation by polarized caveolin-1–rich microdomains, which leads to Ca^2+^ entry, eNOS activation, NO production, and inhibition of NF-κB–mediated transcription. Inhibition of TRPV4 signaling at these domains results in vascular inflammation.

To test whether TRPV4 activation could attenuate the endothelial inflammatory response in vivo, we used a mouse model of acute LPS exposure (1.5 mg/kg). We treated mice with or without GSK101 (10 μg/kg) and isolated the blood and aorta after 4 hours (Figure 10A). LPS treatment led to an increased concentration of inflammatory cytokines in the plasma (Supplemental Figure 11, A and B), a strong upregulation of inflammatory gene expression in EC-enriched RNA from the descending aorta (Supplemental Figure 11, C and D), and substantial VCAM-1 expression on aortic endothelium accompanied by nuclear NF-κB p65 (Supplemental Figure 11E). Cotreatment of animals with GSK101 suppressed LPS-induced aortic endothelial inflammatory gene expression, including that of the NF-κB pathway genes *Rel*, *Rela*, and *Relb* (Figure 10, B and C). Notably, *Nos3* was also increased in animals cotreated with GSK101 (Figure 10B). En face imaging of the descending aortae of animals cotreated with GSK101 also showed a reduction of endothelial VCAM-1 and nuclear NF-κB p65 expression (Figure 10D). In summary, these data show that TRPV4 activation dampens the endothelial inflammatory response in vitro and in vivo.

## Discussion

Our work shows that laminar flow preferentially polarized caveolin-1 to unique signaling domains at the downstream end of arterial ECs. This polarization activated TRPV4 and eNOS in a spatially restricted manner to suppress inflammatory pathways (Figure 10E). Caveolin-1 clustering at the downstream end occurred in response to flow alignment in aortic ECs in vivo and in vitro. We reveal that these clusters define a previously undescribed mechanosensitive domain that enables focal Ca^2+^ entry for eNOS activation and inhibition of inflammatory signaling. These domains were associated with a distinct pattern of proteins, lipids, and consequently biophysical properties rendering the downstream luminal surface of flow-aligned HAECs notably different from other parts of the cell. It remains unclear how elongation and laminar shear stress induce this local modulation of the plasma membrane, with both cytoskeletal and membrane lipids possible mediators (40, 41). Considering that the lipid phosphatidylinositol-4,5-bisphosphate (PIP_2_) has been shown to directly regulate TRPV4 activity in brain capillary endothelium (42, 43), the role of PIP_2_ and other plasma membrane lipids at these domains in response to changes in shear stress remains of interest.

Caveolin-1 and caveolae have been long recognized as important regulators of arterial organization in response to shear stress, in particular with respect to the regulation of vascular tone by both recruitment and modulation of eNOS activity (44–46). Our data indicate that caveolin-1 clustering also plays a role in promoting endothelial cell resilience by dampening inflammatory gene expression. Shear stress has previously been shown to induce caveola formation at the luminal surface of arterial ECs (47, 48); however, polarization of caveolae in this setting has not been shown. Future investigation of how caveolin-1 clustering affects the organization and dynamic redistribution of caveolae and membrane lipid composition will thus be of particular interest, as will be the contribution of the actin cytoskeleton.

Cell polarity is an important regulator of vascular phenotype and function (49). While the apical-basolateral polarity of ECs is clearly defined within the vessel wall, the mechanisms underlying the establishment and maintenance of a lateral polarity in the aorta are poorly understood (50, 51). Intracellular organelles, the Golgi apparatus and the microtubule-organizing center, are often used as indicators of endothelial planar polarity with respect to flow direction (4–6). However, after development, ECs in the descending aorta frequently display the Golgi apparatus positioned at the side of the nucleus (52), indicating neither an upstream nor a downstream polarization. Our current finding of polarized signaling domains, along with other reports of asymmetrically distributed plasma membrane proteins (7–9), suggests that the EC surface may serve as a direct readout of blood flow direction and, therefore, the lateral polarity of the arterial EC.

Cells use the compartmentalization of signaling events to sustain important physiological functions and prevent crosstalk across pathways. For this, they concentrate specific biochemical components to subcellular regions and membranes. Compartmentalization has been described for the cell plasma membrane where membrane components cluster due to specific interactions to achieve spatiotemporal control (11, 12, 53). We show that flow elongation enables the polarized accumulation of caveolin-1 in the endothelial plasma membrane to create lipid-rich domains that compartmentalize Ca^2+^ signaling in the presence of flow. While TRPV4-mediated Ca^2+^ fluxes have been correlated with caveolae microdomains in ECs (54–56), visualization of Ca^2+^ entry after cell alignment with flow, the subcellular polarization of activity with respect to flow direction, and the mechanosensitive nature of the activity have not been clearly shown. By cycling the applied flow on and off, we revealed that Ca^2+^ oscillations were only sustained when laminar shear stress was present, thus indicating the mechanosensitivity of the polarized domain. Importantly, since all imaging experiments were performed after 48 hours of flow exposure, these Ca^2+^ oscillations were associated with the continuous presence of flow, in contrast to previous observations of Ca^2+^ spikes at the onset of shear stress (57, 58). Here, we show that TRPV4 channels at the downstream end of arterial ECs produce localized Ca^2+^ oscillations in the presence of sustained laminar flow. We assert that this compartmentalization of signaling activity is an important feature by which aortic ECs promote pro-vasodilatory and antiinflammatory pathways in the presence of atheroprotective flow.

TRPV4 is a mechanosensitive ion channel (59, 60) shown to associate with endothelial caveolae for Ca^2+^-dependent eNOS activation in pulmonary arteries (61). However, the modulation of its activity in the plasma membrane under flow was less clear. The physical association of TRPV4 with caveolin-1 at the downstream end of the cell indicated that this activation mechanism plays an important role in restricting Ca^2+^ entry to this region of the cell. Since inhibiting the TRPV4-mediated Ca^2+^ influx resulted in reduced NO production and an increase in both ROS and inflammation, our data suggest that endothelial cell resilience is clearly dependent on the presence and activity of these polarized plasma membrane domains. While endothelial TRPV4 activity has been shown to promote vasodilation in arteries (18, 62, 63), its role in vascular inflammation has not been as clearly defined (64). In fact, there are conflicting data regarding the role of TRPV4 activity in the inflammatory cascade (20, 21, 65). Here, we provide evidence that TRPV4 activity under laminar flow is antiinflammatory in aortic ECs. It led to eNOS activation and the production of NO, both indicators of antiinflammatory signaling (66), and ultimately contributed to the dampening of inflammatory gene expression. Therefore, this localized TRPV4 activity is a previously unrealized antiinflammatory mechanism of laminar flow and eNOS activation.

Additional studies are required to determine whether there exists a critical shear stress level or “set point” (67) to initiate and sustain the localized signaling activity. Live cell imaging experiments showed a correlation between EC aspect ratio and the presence of polarized Ca^2+^ activity, suggesting that there is a shear stress “set point” that promotes both cell body elongation and localized signaling activity. It is interesting to note that flow-aligned ECs displayed variation in cell aspect ratio and caveolin-1 clustering, but the underlying contributors to this heterogeneity are unknown.

Recent work has highlighted variable VCAM-1 and CD36 expression in the mouse aorta (68) and polarized VE-PTP clustering in ECs of the mouse aorta and vena cava (69). Additionally, single-cell RNA sequencing data have revealed 5 distinct populations of ECs exposed to laminar flow (70). It is interesting to consider that these populations may be correlated with different levels of cell resilience capacity, as previous work showed that ECs exhibited variable expression levels of VCAM-1 after exposure to an inflammatory agonist (71). Spatial transcriptomics can be applied to correlate the transcriptional signature of ECs with the presence and activity of polarized Ca^2+^ signaling domains. Whether there exists a pattern to the distribution of the ECs with these TRPV4 signaling domains is not yet known. It is intriguing to consider that these signaling cells may act as hubs that are spatially distributed across the endothelium to promote vessel resilience.

Considering that reduced eNOS activity and increased superoxide production are known contributors to EC dysfunction (72, 73), investigation into the role of these polarized caveolin-1/TRPV4 signaling domains in human vascular diseases is warranted. If the presence and activity of these microdomains function as an endothelial resilience mechanism, then diminished domain activity may contribute to EC dysfunction in response to cardiovascular risk factors such as diabetes, dyslipidemia, and aging. Furthermore, since the regulation of eNOS activation is finely tuned (74), identifying the downstream pathways directly affected by these signaling domains will be critical for establishing their role in vascular health and disease. Future research will also establish whether additional protective functions of laminar flow are mediated by polarized caveolin-1/TRPV4 microdomains.

In conclusion, we reveal that the anterior-posterior endothelial cell polarity established in response to laminar flow results in mechanosensitive signaling domains that both promote cell resilience and suppress inflammation.

## Methods

Further information can be found in Supplemental Methods.

### Sex as a biological variable

Our study used both male and female wild-type mice and primary ECs from both male and female donors.

### Cell culture and shear stress

Primary HAECs (Cell Applications S304-05a), immortalized TeloHAECs (ATCC CRL-4052), or primary HUVECs (VEC Technologies lot NCI-1199 pooled from 24 donors) were used. Primary HAECs were used from P4 to P12, and TeloHAECs were used up to P20. Primary HUVECs were used from P5 to P9. For cell culture experiments, MCDB-131 complete medium (VEC Technologies MCDB-131 Complete) or EGM-2 medium (Lonza CC-3162) was supplemented with 10% FBS (Omega USDA certified FBS FB-11). For plating of cells on cell culture dishes, 0.1% gelatin (Stemcell Technologies 07903) coating was first applied. Cells were cultured in a 37°C incubator with 5% CO_2_.

For application of shear stress, ECs were seeded in μ-Slide 0.4 Luer ibiTreat (ibidi 80176) or Y-shaped ibiTreat μ-slides (ibidi 80126). Unidirectional laminar flow was applied to confluent monolayers using the ibidi pump system (ibidi 10902). For experiments requiring access to the cells, including atomic force microscopy, cell monolayers were exposed to shear stress in glass-bottom 6-well plates (Cellvis P06-1.5H-N) or 35 mm glass-bottom FluoroDishes (WPI FD35-100) on an orbital shaker (Benchmark Scientific BT302). A rotation speed of 130 rpm was applied to achieve endothelial cell alignment on the periphery of the well where the flow is unidirectional, while cells were unaligned in the center of the well where flow is multidirectional (75, 76). For cell alignment in the absence of flow, cells were plated on customized ibidi μ-slides with micropattern surface (ibidi 83851).

### Live cell fluorescence imaging

#### Membrane fluidity.

For visualization of cell plasma membrane fluidity, Laurdan dye (6-dodecanoyl-2-dimethylaminonaphthalene) (Invitrogen D250) was applied at 10 μM to cell monolayers and incubated 30 minutes at 37°C, then washed and imaged in 1× HBSS. For imaging, Zeiss LSM 880 with Chameleon 2-photon laser with Plan-Apochromat ×20/0.8 M27 objective and GaAsP photomultiplier tube (PMT) array detector were used. Images were collected using 2-photon excitation set to 770 nm, and 2-channel emission detection was set for 400–460 nm and 470–530 nm. The acquisition of generalized polarization images was performed using ImageJ software (NIH) and ImageJ macro GPCalc ratiometric quantification and visualization (77).

#### Lipid raft imaging.

Live cell imaging was performed using BODIPY FL C_5_-Ganglioside G_M1_ (Invitrogen B13950). Cell monolayers were treated with 50 μM BODIPY FL C_5_-Ganglioside G_M1_ for 20 minutes at 37°C with Hoechst 33342 (AAT Bioquest 17535).

#### Calcium imaging.

HAECs and HUVECs were transfected with GCaMP plasmid (pPB_CAG_GCamp5g) with PiggyBac construct (PB_Vector) (gift from R. Wollman, UCLA, Los Angeles, California, USA) using Lipofectamine 3000 transfection reagent (Invitrogen L3000001) or TransIT-X2 Dynamic Delivery System (Mirus MIR6010) in Opti-MEM medium (Gibco 31985062) overnight. Cells were then selected for GCaMP expression with blasticidin (Gibco A1113903). GCaMP-expressing HAECs were plated on Y-shaped chamber μ-slides (ibidi 80126), and after reaching confluence, slides were connected to the ibidi pump system for flow conditioning. After 48 hours of unidirectional flow, the Y-slide was connected to Yellow/Green perfusion set (ibidi 10964) modified with non-permeable tubing containing 13 mL of conditioned MCDB-131 (VEC Technologies MCDB-131 WOFBS) with 10% FBS (Omega USDA certified FBS FB-11) for live cell imaging. All steps were completed with minimal light exposure. Fluorescence images were acquired using Zeiss Observer Z1 with Colibri 7 light source, CMOS camera (Photometrics Prime 95B), and ZEN Blue software. Images were acquired once every 3 seconds for total imaging time. For the long-term live cell imaging of the flow alignment, the ibidi stage-top incubator system (ibidi 1270) was used in combination with μ-slide 0.2 Luer ibiTreat (ibidi 80166) and Yellow/Green perfusion set (ibidi 10964). For observation of changes in calcium activity, chemicals were added to one of the ibidi syringe reservoirs when air pressure was not active. Chemicals used included ethylene glycol tetraacetic acid (EGTA; 1.6 mM; Fisher Scientific NC1280093), Adenosine A1 Receptor Agonist Cyclopiazonic Acid (CPA; 50 μM; Sigma-Aldrich 119135), M-Theraphotoxin-Gr1a (GsMTx4, 5 μM; Alomone Labs STG-100), Yoda1 (0.5 μM; Tocris 5586), GSK1016790A (10 nM or 1 μM; Sigma-Aldrich 530533), and GSK205 (20 μM; MedChemExpress HY-120691A).

#### Nitric oxide imaging.

For quantification of NO production, cells were treated with 5 μM DAF-FM (Invitrogen D23844) for 20 minutes at 37°C with Hoechst 33342 (AAT Bioquest 17535).

#### Reactive oxygen species imaging.

For oxidative stress detection, cell monolayers were treated with 5 μM CellROX Deep Red Reagent (Invitrogen C10422) or 5 μM CM-H_2_DCFDA (Molecular Probes, C6827) for 20 minutes at 37°C with or without Hoechst 33342 (AAT Bioquest 17535).

#### Membrane cholesterol imaging.

pALOD4 was a gift of A. Radhakrishnan (UT Southwestern Medical Center; Addgene plasmid 111026) (78), and ALOD4 was purified and labeled with Alexa Fluor 488 as previously described (79, 80). Visualization of the accessible pool of cholesterol was performed after 30 minutes of treatment with 10 mM methyl-β-cyclodextrin (MβCD) in 1% lipoprotein-deficient serum (LPDS; Sigma-Aldrich S5519) or 1% LPDS control, followed by flow for 2 hours in complete medium. HAECs were then stained with 20 μg/mL ALOD4-488 for 10 minutes at room temperature followed by 4% paraformaldehyde (PFA; Sigma-Aldrich 322415) fixation and application of 4,6-diamidino-2-phenylindole, dihydrochloride (DAPI) nuclear stain (AAT Bioquest 17507).

After respective dye incubation, cell monolayers were washed with 1× HBSS, then imaged in culture medium or 1× HBSS using Zeiss Colibri LED plus CMOS camera (Photometrics 95B) detection or LSM 900 with Airyscan2 GaAsP-PMT detector for gentle confocal imaging.

### Animals

Both male and female wild-type C57BL/6J mice were purchased from The Jackson Laboratory (JAX:000664). All mice were fed a chow diet and water ad libitum under a 12-hour light/12-hour dark cycle.

### Aorta en face immunostaining

Mice were perfused with ice-cold 1× PBS with an incision of the right atrium to release the blood followed by perfusion with 4% PFA. The thoracic aorta was isolated and postfixed with 0.4% PFA overnight at 4°C. The vessel was then washed 3 times with 1× PBS and permeabilized by incubation with 0.3% Triton X in 2% normal donkey serum (Jackson ImmunoResearch Laboratories 017-000-121) in 1× PBS for 30 minutes at room temperature followed by incubation with primary antibodies overnight at 4°C with gentle agitation. After 1× PBS washes, the vessel was incubated with corresponding secondary antibodies for 2 hours at room temperature followed by washing with 1× PBS 3 times for 10 minutes each. The vessel was then cut longitudinally and mounted in Fluoromount-G (SouthernBiotech 0100-01). Primary antibodies used included ERG (Abcam ab92513), VE-cadherin (R&D AF1002), caveolin-1 (Invitrogen PA1-064), cavin-1 (Abcam ab48824), NF-κB p65 (Cell Signaling 8242S), and VCAM-1 (BD Pharmingen 550547).

### Immunofluorescence and confocal imaging

For immunostaining, cell monolayers were fixed with 4% PFA for 10 minutes followed by multiple washes with 1× PBS. Samples were then blocked for 2 hours with 10% normal donkey serum (Jackson ImmunoResearch Laboratories 017-000-121) in 1× PBS. Depending on the protein targets of interest, samples were permeabilized with either 0.1% Triton X-100 (Fisher Scientific A16046-0F) or 0.01% digitonin (EMD Millipore 3004100). Primary antibodies were incubated overnight at 4°C in blocking buffer and secondary antibodies applied for 2 hours at room temperature. Primary antibodies used included caveolin-1 (Invitrogen PA1-064, R&D AF5736, or Santa Cruz Biotechnology sc-894), cavin-1 (Abcam ab48824), caveolin-2 (Invitrogen PA5-21927), ICAM-1 (Santa Cruz Biotechnology sc-107), E-selectin (Invitrogen MA1-06506), ZO-1 (Invitrogen MA3-39100-A488), NF-κB p65 (Cell Signaling Technology 8242S), p-eNOS^Ser1177^ (Invitrogen PA5-35879 or Santa Cruz Biotechnology sc-81510), TRPV4 (Alomone Labs ACC-034 or LSBio LS-C401108), and VE-cadherin (R&D AF938). For F-actin staining, Alexa Fluor 555 Phalloidin (Invitrogen A34055) was applied with secondary antibody incubation.

Imaging was performed on a Zeiss LSM 900 confocal microscope equipped with 405 nm, 488 nm, 561 nm, and 640 nm laser lines using Plan-Apochromat objectives (×10, ×20, ×40, or ×63) and Airyscan2 GaAsP-PMT detector. Identical laser intensity settings were applied to all samples being compared with equivalent *Z* thickness. After acquisition, a maximum-intensity projection of the *Z*-stack was applied using ZEN Blue 3.5 software (Zeiss). Image processing and quantification of parameters were performed with ZEN Blue software, IMARIS software (Bitplane), ImageJ (NIH), or custom script (see *Image analysis* section in Supplemental Methods for details).

### Drawings

Schematics in Figure 1B; Figure 2, B and C; Figure 3, A and B; Figure 4, A and C; Figure 5, A and B; Figure 6, A, E, and F; Figure 7A; Figure 8D; Figure 10, A and E; Supplemental Figure 1, A–C and E; Supplemental Figure 2A; Supplemental Figure 3A; and Supplemental Figure 4, A and B were created with BioRender (biorender.com).

### Statistics

Statistical analysis was performed using GraphPad Prism software. The results are presented as mean ± SD. Depending on how many conditions were compared, either 2-tailed independent *t* test analysis or 1-way ANOVA with Tukey’s post hoc multiple-comparison test was conducted. The Pearson’s correlation coefficient (*r*) was used to measure the strength of a linear association between 2 variables. *P* < 0.05 was considered statistically significant for all analyses.

### Study approval

All mouse experiments were approved by the Institutional Animal Care and Use Committee and Animal Review Committee at UCLA.

### Data availability

Values for all data points in graphs are reported in the Supporting Data Values file. Sequencing data were deposited in the NCBI’s Gene Expression Omnibus database (GSE255770). Custom code used for image analysis is available via a public GitHub repository link: https://github.com/marcusgj13/endoSeg

## Author contributions

JJM initiated, designed, and supervised the study. SGH, JWA, JPK, EC, MW, MS, and JJM performed experiments. RF, P Tontonoz, EJT, and P Turowski shared reagents and experimental expertise. MGJ wrote analysis code and performed image analysis. P Turowski and JJM wrote the paper.

## Supplementary Material

Supplemental data

Unedited blot and gel images

Supplemental video 1

Supplemental video 2

Supplemental video 3

Supplemental video 4

Supplemental video 5

Supplemental video 6

Supplemental video 7

Supplemental video 8

Supplemental video 9

Supplemental video 10

Supplemental video 11

Supplemental video 12

Supporting data values

## Version 1 05/21/2024

In-Press Preview

## Version 2 07/01/2024

Electronic publication
