# Supplementary material for: Mechanosensitive membrane domains regulate calcium entry in arterial endothelial cells to protect against inflammation
Source: J Clin Invest. 2024 May 21;134(13):e175057. doi: 10.1172/JCI175057 (PMC11213468; doi:10.1172/JCI175057)
Supplement: Supplemental data [file jci-134-175057-s266.pdf]

Supplemental Materials

Mechanosensitive membrane domains regulate calcium entry in arterial endothelial cells to protect against inflammation

Authors: Soon-Gook Hong, Julianne W. Ashby, John P. Kennelly, Meigan Wu, Michelle Steel, Eesha Chattopadhyay, Rob Foreman, Peter Tontonoz, Elizabeth J. Tarling, Patric Turowski, Marcus Gallagher-Jones, and Julia J. Mack

This file contains the following:
Supplemental Methods
Supplemental References
Supplemental Figures 1 – 11
Supplemental Videos

Supplemental Methods

Atomic force microscopy (AFM)

Surface measurements were performed on HAECs using JPK NanoWizard 4a AFM and Bruker PFQNM-Live Cell probe with a spherical tip (Bruker AFM Probes #PFQNM-LC) and tip length 17 µm and tip radius 65 nm. The spring constant of the cantilever was determined by manufacturer and inputted into JPK acquisition software. Imaging was performed using QI™ mode for quantitative imaging to capture the entire cell and plot the height profile. Force mapping was then performed at the two ends of the cell as user defined 15 x 32 pixel regions with tapping frequency of 10 Hz and probe extend speed of 3 µm/sec and 0.6 nN setpoint applied. To quantify the Young's modulus, JPK Data Processing analysis software was used to fit individual force curves using Hertz model. For identification of the cell surface properties, the indentation depth was set to less than 100 nm for each force curve to keep within 0.1% strain.

Proximity ligation assay

The Duolink proximity ligation assay (PLA) was performed according to the manufacturer's protocol (Sigma Aldrich #DUO92008). Briefly, HAECs were grown on Y-shaped chamber slides (ibidi #80126) and after reaching confluence, slides were connected to ibidi pump system for flow (20 dynes/cm^2, unidirectional laminar flow) for 48 h. Then, cells were fixed with 4% PFA for 5 min at room temperature. After washing with 1X PBS, the cells were permeabilized with 0.05% Triton X-100 (Fisher Scientific #A16046-0F) in 1X PBS for 5 min, blocked in Duolink blocking solution for 60 min at 37˚C and then incubated with primary antibody (anti-TRPV4, LSBio #LS-C401108 and anti-Caveolin-1, R&D #AF5736; anti-TRPV4 or anti-Caveolin-1 only; anti-Caveolin-1 and anti-

nuclear HistoneH3 Abcam #ab8898, as negative controls; anti-Caveolin-1 and anti-Cavin-1, Abcam #ab48824, as a positive control) diluted in Duolink Antibody Diluent overnight at 4˚C. After washing, the cells were incubated with PLA probes including PLUS-Goat (#DUO92003), MINUS-Rabbit (#DUO92005) at 37˚C for 1 h. After ligation, amplification, and final washes, the cells were counter-stained with ZO-1 conjugated with AF488 (Invitrogen #MA3-39100-A488) and DAPI (AAT Bioquest #17507). Images were obtained using Zeiss LSM900 with Airyscan2 GaAsP-PMT detector.

Image analysis

For fluorescence intensity measurements, maximum intensity projections of the confocal Z-stack images were used. To quantify intensity differences with treatments for ALO-D4, DAF-FM, CellROX, ICAM-1, E-Selectin and TRPV4, the arithmetic mean intensity for each channel of interest was determined using ZEN Blue software (Zeiss, Germany). For nuclear quantification of NF-κB p65 localization, ImageJ was used to create a binary mask on the DAPI stained nuclear channel. The nuclear mask was then applied to the NF-κB p65 stained channel using the ROI Manager and the mean fluorescence intensity was calculated per cell. For graphical tracing of the subcellular Ca^{2+} activity, the GCaMP intensity over time was exported using ZEN Blue software using the ROI feature for each defined region of interest. Extracted data was plotted in GraphPad Prism software.

For analysis of the live cell GCaMP activity and confocal imaging, segmentation was performed by hand-drawing cell borders to input the cell segmentation mask and raw image file into custom code endoSeg, see section below for code description, designed to extract cell features and fluorescence intensity characteristics at subcellular regions. Cell border masks were created using Procreate® software, version 5.3.2, for iPad (Apple

Inc.). Each mask was hand-drawn as a continuous brush stroke for all full-length cells in the field of view (see example in Supplemental Figure 1C).

For visual identification of the calcium activity location in HAECs, the “Time differential” function in ZEN Blue 3.5 software was used to view location of fluorescence intensity changes and, therefore, sites of Ca^{2+} spikes. The original file and resulting file from the time differential were processed using “Maximal Projection” function in ImageJ, and the two images merged.

To quantify the physical association of Caveolin-1 and TRPV4, the number of PLA puncta per cell were counted. Briefly, images were processed using ImageJ (NIH) to subtract background and perform binary conversion. After binary conversion, PLA puncta were identified and counted using the ‘Analyze particles’ function.

Caveolin-1 cluster analysis was achieved by quantifying Caveolin-1 staining within individual cells as identified by VE-Cadherin boundaries. Each cell was further segmented into equal length thirds for the ‘upstream’, ‘mid-body’ and ‘downstream’ regions. Confocal images were processed using ImageJ (NIH) for binary conversion. Caveolin-1 clusters were quantified by measuring the number of particles and total area coverage using the ‘Analyze particles’ function. The cluster index was then calculated using the following equation:

$$Cluster\ Index\ = \frac{Total\ Area\ Coverage}{Total\ Number\ of\ Particles}$$

endoSeg

To quantify the localization of fluorescence signal in cells, we employed a series of custom analysis routines, implemented in Python, termed endoSeg. First, a border mask is

required to define the boundaries of all cells within the particular field of view (FOV). The mask is binarized using Otsu's method and trimmed. Following segmentation, individual cells are passed through one of two separate but related analysis pipelines depending on whether the input data are multi-channel fluorescence images or time-series Ca^{2+} tracking images.

For multi-channel image sets, the individual cell masks from the watershed segmentation are used to calculate the orientation of the cell as well as additional parameters such as the centroid, area and aspect ratio. The centroid and orientation are then used to create a map that computationally splits the cell into three equally sized sectors, an upstream third, a middle third and a downstream third. The total fluorescence signal of the whole cell is then normalized to be zero mean and standard deviation one to remove the influence of cell-to-cell variation in absolute fluorescence intensity. The remaining negative values are set to zero. For each fluorescent channel of interest, the mean, maximum and Index of Dispersion (IoD) of the signal are calculated for each sector and recorded. Since the IoD is the ratio of the variance to the mean, the presence of a polarized signal is reported in the output based on the IoD value being over two in a specific region.

For time-series image sets, the analysis starts similarly, with orientation and sector maps being calculated. For each time-series, the mean fluorescence intensity is calculated from the whole cell and for each of the three sectors to give a one-dimensional plot of fluorescence intensity over time. The decay of fluorescence intensity due to bleaching is corrected using a Savitzy-Golay filter with a window size of 51 pixels and a 5th-order polynomial. These particular values were selected to produce a smoothed representation

of the signal decay while ignoring the presence of intensity spikes to avoid over-subtraction. Following the intensity decay correction, a median filter is applied to remove any remaining spurious background and negative values are set to zero. The mean, maximum and IoD of the fluorescence time-series are calculated for the whole cell and each of the three sectors. We found that the IoD can be quite sensitive to the absolute fluorescence intensity of a particular cell. To make this value comparable across all cells within a given FOV, the IoD for each sector is divided by the IoD of the whole cell. This ratio of IoD is then used to determine the presence of intensity spikes (representing rapid changes in fluorescent signal) with a value greater than 2 being used as the threshold. Cell aspect ratio analysis and visualisation was performed in Python using the scikit-image library. In brief, individual masks were segmented in endoSeg and the orientation, long and short axes of the segmented cells were determined using scikit-image's measure region properties function. The scripts for creating the images can be found within the endoSeg repository.

RNA sequencing

HAECs were seeded on 6-well plates and subjected to flow using an orbital shaker with a rotation speed of 130 rpm for 48 h in a 37°C incubator with 5% CO_2. After 48 h, the flow-conditioned HAEC monolayers were treated with either DMSO or GSK205 (20 µM; MedChemExpress #HY-120691A) for 2 h under flow. Prior to cell lysis, non-aligned cells from the center of each well were removed using 1.8 cm blade cell scraper (Falcon #353085) and washed with 1X DPBS (Gibco #14190-144). The remaining flow-aligned cell monolayer was lysed using RLT lysis buffer (QIAGEN #79216) plus 1% 2-Mercaptoethanol (Sigma Aldrich #M6250). For each condition, 2 wells were combined

as one flow-aligned sample for a total of 6 wells for n = 3 samples. RNA extraction, library construction, and sequencing were conducted by the Technology Center for Genomics & Bioinformatics (TCGB) core facility at UCLA.

Briefly, libraries for RNA-Sequencing were prepared using KAPA Stranded mRNA-Seq Kit (KAPA Biosystems #KK8421). The workflow consists of mRNA enrichment and fragmentation, first strand cDNA synthesis using random priming followed by second strand synthesis converting cDNA:RNA hybrid to double-stranded cDNA (ds-cDNA), and incorporates dUTP into the second cDNA strand. cDNA generation is followed by end repair to generate blunt ends, A-tailing, adaptor ligation and PCR amplification. Different adaptors were used for multiplexing samples in one lane. Sequencing was performed on Illumina Novaseq 6000 for PE 2x100bp run. Data quality check was done on Illumina SAV. Demultiplexing was performed with Illumina Bcl2fastq v2.19.1.403 software. The alignment was performed using Spliced Transcripts Alignment to a Reference (STAR) (1) using the human reference genome assembly GRCh38. The Ensembl Transcripts release GRCh38.107 GTF was used for gene feature annotation. For normalization of transcripts counts, counts per million (CPM) normalized counts were generated by adding 1.0E-4 followed by CPM normalization. RNA sequencing analysis

Differential gene expression was determined by the Partek gene-specific analysis algorithm (GSA; Partek Flow) and pathway enrichment was analyzed using ShinyGO (2). For graphical display of gene expression, Heatmapper (3) was used to create a heatmap of gene expression. Average linkage was used as Clustering Method and Kendall’s Tau was used as Distance Measurement Method. VolcaNoseR was used to

generate the Volcano plot of gene expression levels. Log_2 (fold change; FC) as X-variables and –log10(p-value) as Y-variables were used. ShinyGO (2) was used for gene enrichment analysis with false discovery rate (FDR) of 0.05 as cut-off.

Ingenuity pathway analysis (IPA)

IPA upstream regulator analysis (QIAGEN) was conducted to computationally predict the putative upstream transcriptional regulators that contribute to the gene expression changes. Differentially expressed genes (DEGs) at a p-value cut off of $p < 0.05$ were used for the analysis. The putative upstream regulators were predicted by activation Z-score and a p-value of overlap based on a significant overlap between differentially expressed genes in the dataset and known targets regulated by transcriptional regulators stored in the Ingenuity Knowledge Base.

Gene set enrichment analysis (GSEA)

GSEA 4.3.2 software was used for the analysis. Genes used for GSEA were those that were differentially expressed in GSK205 treatment condition versus DMSO. The Hallmarks gene set database was used with the following analysis parameters: Number of permutations: 1000; Collapse/Remap to Gene Symbols: No Collapse; Permutation type: Gene Set; Chip Platform: Human Gene Symbol with Remapping MSigDB.v2023.1.Hs.chip; Enrichment Statistic: Weighted; Metric for Ranking Genes: Signal2Noise; Gene List Sorting Mode: Real; Gene List Ordering Mode: Descending; Max Size: Exclude Larger Sets: 500; Min Size: Exclude Smaller Sets: 15.

Immunoblotting

To measure protein expression levels, HAEC monolayers were lysed in RIPA buffer (Fisher Scientific #PI89900) with Halt™ Protease and Phosphatase Inhibitor Cocktail

(Thermo Scientific #78840) and 0.1 mM phenylmethylsulfonyl fluoride (PMSF) serine protease inhibitor (Fisher Scientific #PI36978). Lysates were then centrifuged at 16,000g for 15 min at 4°C. Supernatants were collected and subjected to BCA protein assay (Pierce™ BCA Protein Assay Kit #23225) to quantify protein concentrations. Protein samples were run via sodium dodecyl sulphate–polyacrylamide gel electrophoresis (SDS-PAGE) and transferred to a polyvinylidene difluoride (PVDF) membrane using BioRad Trans-Blot® Turbo™ Transfer system (BioRad #1704150) and Trans-Blot® Turbo Mini PVDF transfer packs (BioRad #1704156). Subsequently, the membrane was incubated with 5% non-fat dry milk in Tris-buffered saline with Tween 20 (TBST) for 20 min at room temperature and then incubated overnight at 4°C with respective primary antibodies. The membrane was washed with TBST and incubated with HRP-conjugated secondary antibodies for 1 h. After washing, the membrane was subjected to chemiluminescence method for visualization using ChemiDoc™MP Imaging System (BioRad #17001402). Primary antibodies used for immunoblotting were as follows: anti-TRPV4 (Alomone Labs #ACC-034), anti-Total-eNOS (Santa Cruz #sc-376751), anti-phospho-eNOS^{Ser1177} (Invitrogen #PA5-35879), anti-Caveolin-1 (Santa Cruz #sc-70516), anti-GAPDH (Cell Signaling #2118S), anti-β-Actin (Sigma-Aldrich #A5316).

In vitro inflammatory response

To test the role of TRPV4 activity on EC inflammatory response, HAECs were seeded into ibidi 0.4 μ-slides (ibidi #80176) and subject to flow using ibidi Pump System (ibidi #10902) for 48 h (unidirectional laminar flow, 20 dynes/cm^2) then removed from flow. The flow-aligned HAECs were treated with the pro-inflammatory cytokine, TNFα (10 ng/mL; R&D Systems #210-TA-020/CF) in the presence of DMSO or GSK1016790A (10nM;

Sigma-Aldrich #530533) for 30 min. Inflammatory phenotype was characterized by quantifying NF-κB p65 nuclear localization and ICAM-1 expression as well as inflammatory gene expression of *NFKBIA*, *VCAM1*, *CCL2*, *SELE*, and *TNF*.

Transfection with small interfering RNA (siRNA)

HAECs were transfected with either stealth siRNA targeting *TRPV4* (si*TRPV4*, Invitrogen #HSS126973) or negative control stealth siRNA Med GC Duplex #2 (Thermo Fisher #12935112) using TransIT-X2 Dynamic Delivery System (Mirus #MIR6000) in Opti-MEM® Reduced Serum Medium (Gibco #31985070) without antibiotics according to the manufacturer's guidelines. In order to improve transfection efficiency, HAECs were transfected twice with a 24 h recovery in between, then exposed to flow within 24 h after the second transfection.

Gene expression analysis

For qPCR analysis, total RNA was extracted from adherent cells using RNeasy Mini kit (QIAGEN #74104). RNA was converted to cDNA by reverse transcription and cDNA was quantified using a Nano-Drop 8000 (Thermo Fisher). Target genes were quantified using iTaq Universal SYBR Green Supermix (BioRad #1725125) and the appropriate primer pairs. Samples were run on a QuantStudio 6 Flex 384-well qPCR apparatus (Applied Biosystems). Each target gene was normalized to *HPRT*. Primer sequences (5′ – 3′) are listed below:

Gene	Forward Primer	Reverse Primer
KLF2	CATCTGAAGGCGCATCTG	CGTGTGCTTTCGGTAGTGG
KLF4	AGAGTTCCCATCTCAAGGCA	GTCAGTTCATCTGAGCGGG
TRPV4	GGTGGATGAGGTGAACTGGT	GGTAGGTCTCATTCTTGCCCG

NFKBIA	TCAACAGAGTTACCTACCAGGGC	TCCTCTGTGAACTCCGTGAACTCT
VCAM1	GGAATTAACCAGGCTGGAAGA	TGTCTCCTGTCTCCGCTTTT
CCL2	CCCCAGTCACCTGCTGTTAT	AGATCTCCTTGGCCACAATG
SELE	AAGGCTTCATGTTGCAGGGA	ATTCATGTAGCCTCGCTCGG
TNF	AGCCCATGTTGTAGCAAACC	TGAGGTACAGGCCCTCTGAT
CD31	GAAACCATGCAATGAAACCA	GACAGCTTTCCGGACTTCAC
ITGB1	TTATTGGCCTTGCATTACTGCT	CCACAGTTGTTACGGCACTCT
HPRT	GCCCTGGCGTCGTGATTAGT	AGCAAGACGTTCAGTCCTGTC

In vivo injections

Wildtype C57BL/6J mice (JAX:000664) were injected with GSK205 (10 mg/kg), lipopolysaccharides from *Escherichia* coli serotype O111:B4 (LPS at 1.5 mg/kg dose diluted in 1X PBS; Sigma-Aldrich #L2630), DMSO and/or GSK101 (10 µg/kg; Sigma-Aldrich #530533) via 31g x ½" needles via intraperitoneal (IP) injection and fasted until harvest. Female mice were used for the experiment with GSK205 and male mice were used for the LPS inflammatory stimulus experiment. For the sac, the mice were heavily anesthetized with isoflurane and blood was collected from the retro-orbital sinus. Death was confirmed afterwards by cervical dislocation. The mouse was then opened for perfusion with 1X DMEM (Corning #10013CV) followed by aorta isolation.

Aortic endothelial gene expression

Aortas were removed and fat cleaned from vessel exterior in 1X DMEM (Corning #10013CV). The lumen of the descending aorta was flushed with 100 µL RLT lysis buffer (QIAGEN #79216) plus 1% 2-Mercaptoethanol (Sigma Aldrich #M6250) via 30 gauge blunt needle (Strategic Applications Inc. #B3050). RNA was isolated using RNeasy Micro Kit (QIAGEN #74004). For qPCR analysis, RNA was converted to cDNA by reverse

transcription and cDNA was quantified using a Nano-Drop 8000 (Thermo Fisher). Target genes were quantified using iTaq Universal SYBR Green Supermix (BioRad #1725125) and the appropriate primer pairs. Samples were run on a QuantStudio 6 Flex 384-well qPCR apparatus (Applied Biosystems). Each target gene was normalized to *Hprt*. Primer sequences (5′ – 3′) are listed below:

Gene	Forward Primer	Reverse Primer
Hprt	CTGGTTAAGCAGTACAGCCCCAA	CGAGAGGTCCTTTTCACCAGC
Trpv4	TTCACCCTCACCGCCTACTA	AGCCTCAGGTAGTCCACTGT
Cdh5	AAGCTGCCAGAAAACCAGAA	AATCGGAAGAATTGGCCTCT
Pecam1	AGCCTAGTGTGGAAGCCAAC	TTCCGTTCTCTTGGTGAGGC
Nos3	CTGCAAACCGTGCAGAGAATT	CACCGGCTTCATCCAGCT
Klf2	ACCAAGAGCTCGCACCTAAA	GTGGCACTGAAAGGGTCTGT
Klf4	GTGCCCCGACTAACCGTTG	GTCGTTGAACTCCTCGGTCT
Cav1	CACACCAAGGAGATTGACCTGG	CCTTCCAGATGCCGTCGAAACT
Cavin1	GCGCCGCAACTTCAAAGTCATG	TCACCCTCCTTCTCAGGCAGTG
Rel	GCTGCAAGATTGTGGTCACTT	ATCTTCCTTCTCCAGTTGATCCC
Rela	TGAGCCCATGGAGTTCCAGTA	GGTCCTTTTGCGCTTCTCTTC
Relb	CGGTGTGGACAAGAAGCGAA	CGTTTGCTCTCGATTCCATGTG
Ikkbk	CTGCTTCAGGCAATCCAAAGC	CCACGGTCTTACTGAGCTGT
Nfkbia	TGCACCTAGCCTCTATCCAC	CGTTGACATCAGCACCCAAAG
Ccl2	TTCACCAGCAAGATGATCCCA	ACCTCTCTCTTGAGCTTGGTG
Icam1	CCCACGCTACCTCTGCTC	GATGGATACCTGAGCATCACC
Vcam1	TTCACGTGGGGCACAAAGAA	AGCTTGAGAGACTGCAAACAGTA
Sele	GGACACCACAAATCCCAGTCT	TCGCAGGAGAACTCACAACTG
Selp	AAGATGCCTGGCTACTGGACAC	CAAGAGGCTGAACGCAGGTCAT

Tnf	CTGTAGCCCACGTCGTAGC	TTTGAGATCCATGCCGTT
Il1b	TTTGAAGCTGGATGCTCTCAT	AGTTGACGGACCCCAAAAG

Cytokine analysis

Plasma samples were sent to the UCLA Immune Assessment Core for standardized multiplex cytokine Luminex testing. Samples were run on the Luminex mouse 32-plex cytokine/chemokine panel using the Luminex 200 system.

Supplemental References

1. Dobin A, Davis CA, Schlesinger F, Drenkow J, Zaleski C, Jha S, et al. STAR: ultrafast universal RNA-seq aligner. *Bioinformatics.* 2013;29(1):15-21.
2. Ge SX, Jung D, and Yao R. ShinyGO: a graphical gene-set enrichment tool for animals and plants. *Bioinformatics.* 2020;36(8):2628-9.
3. Babicki S, Arndt D, Marcu A, Liang Y, Grant JR, Maciejewski A, et al. Heatmapper: web-enabled heat mapping for all. *Nucleic Acids Res.* 2016;44(W1):W147-53.

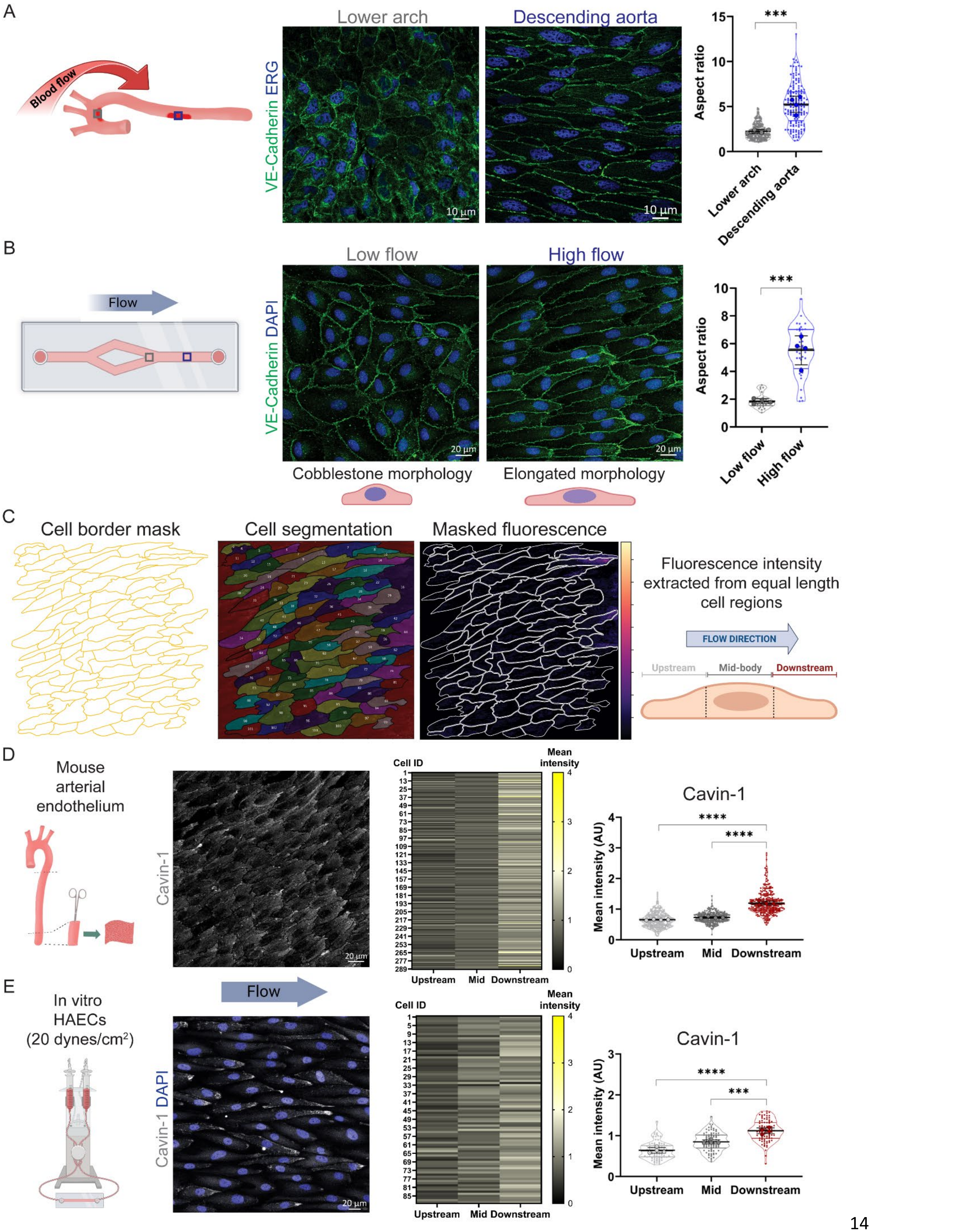
A
Blood flow
Lower arch
Descending aorta
VE-Cadherin ERG
10 µm
10 µm
Aspect ratio
15
10
5
0
***
Lower arch
Descending aorta
B
Flow
Low flow
High flow
VE-Cadherin DAPI
20 µm
20 µm
Cobblestone morphology
Elongated morphology
Aspect ratio
10
8
6
4
2
0
***
Low flow
High flow
C
Cell border mask
Cell segmentation
Masked fluorescence
Fluorescence intensity extracted from equal length cell regions
FLOW DIRECTION
Upstream
Mid-body
Downstream
D
Mouse arterial endothelium
Cavin-1
20 µm
Cell ID
Mean intensity
Upstream
Mid
Downstream
Cavin-1
Mean intensity (AU)
****
****
Upstream
Mid
Downstream
E
In vitro HAECs (20 dynes/cm²)
Flow
Cavin-1 DAPI
20 µm
Cell ID
Mean intensity
Upstream
Mid
Downstream
Cavin-1
Mean intensity (AU)
****
***
Upstream
Mid
Downstream

Supplemental Figure 1: High flow induces arterial endothelial cell elongation and polarization of plasma membrane associated protein Cavin-1.

(**A**) Representative images from en face imaging of the endothelium from a wildtype mouse aorta stained for VE-Cadherin and ERG. Border masks were applied to calculate cell aspect ratio for 215 and 166 cells in the lower arch and in the descending aorta, respectively, from n = 4 aortas. ***$P < 0.001$ by two-tailed, unpaired *t* test. (**B**) HAECs were seeded on y-shaped chambers and exposed to laminar flow for 48 h, then fixed and stained for DAPI and junctional marker VE-Cadherin. Aspect ratio was quantified for cells in low-flow (~5 dynes/cm^2; n = 28) and high-flow (~20 dynes/cm^2; n = 38) regions from n = 4 biological replicates. ***$P < 0.001$ by two-tailed, unpaired *t* test. (**C**) Process of cell segmentation to extract fluorescence intensity from subcellular regions. Cell border mask is inputted in endoSeg to generate monolayer segmentation with cell IDs, masked fluorescence overlay, and values for 'upstream,' 'mid-body,' and 'downstream' segments for each cell. (**D**) Confocal imaging of Cavin-1 in the endothelium of wildtype mouse descending aorta. Individual cells were segmented into 3 equal-length regions (upstream, mid and downstream), and the staining intensity was determined for each segment in 289 cells from n = 4 aortas. Bar graph displays the mean ± SD with data analyzed by one-way ANOVA and post hoc Tukey's multiple comparisons test. ****$P < 0.0001$. (**E**) As for (D) but using n = 88 flow-aligned HAECs from 4 biological replicates. ***$P < 0.001$, ****$P < 0.0001$ by two-tailed, unpaired *t* test.

Supplemental Figure 2

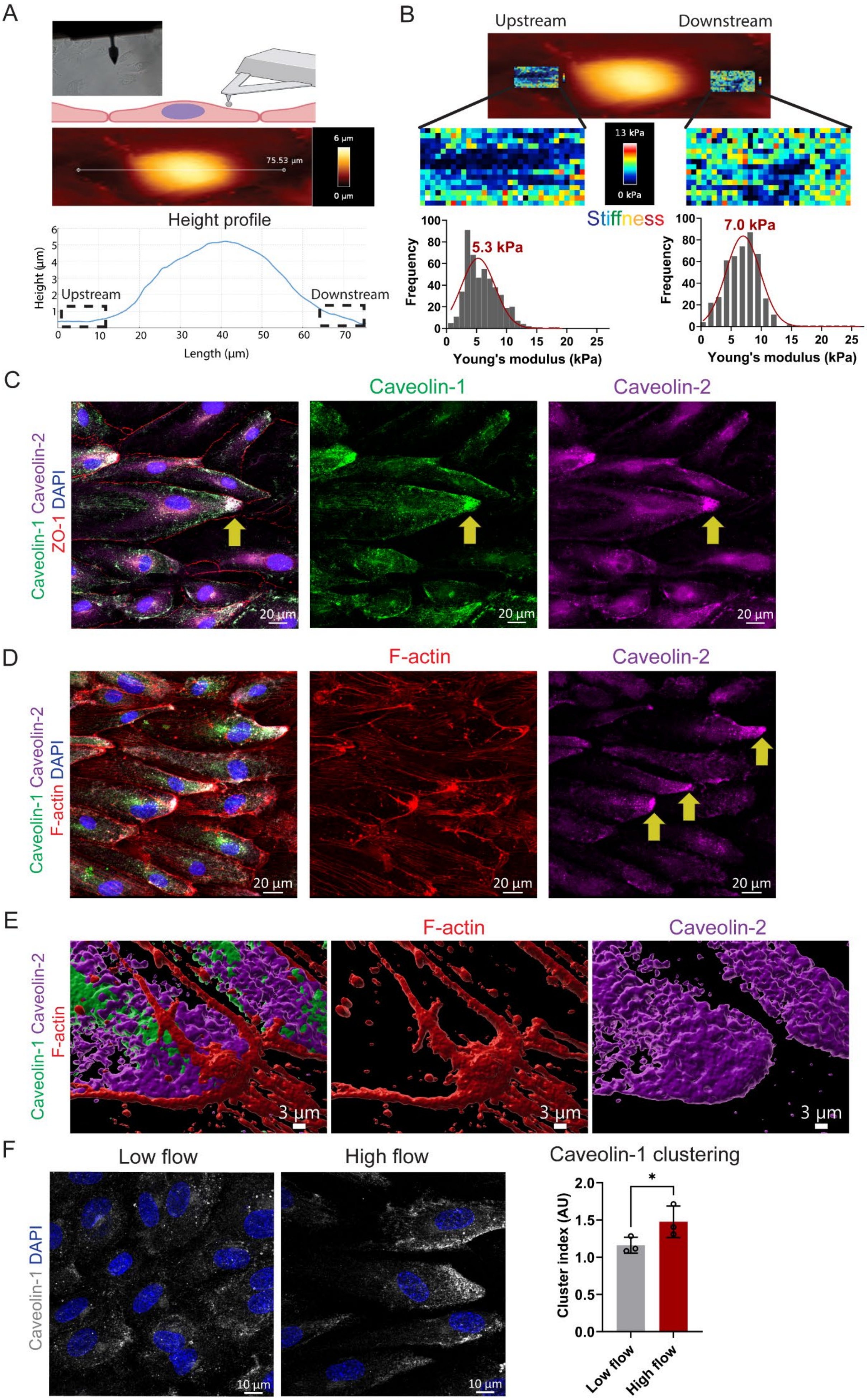

Supplemental Figure 2: Features of high flow induced asymmetry of the cell surface.

(**A**) Atomic force microscopy (AFM) of flow-aligned HAEC surface: shown is the experimental setup with phase contrast image and the height profile of a representative cell. Upstream and downstream ends were clearly discernible. (**B**) Cell stiffness was measured by calculating the Young's modulus (kPa) from the force curves acquired at the upstream and downstream regions. The Young's modulus values are displayed as a frequency plot with mean value for each region. (**C**) Confocal imaging of flow-aligned HAECs displays Caveolin-1 and Caveolin-2 protein at the downstream end of cell as marked by yellow arrow. (**D**) Representative confocal images of flow-aligned HAECs showing Caveolin-2 protein accumulation at the downstream end of cells where F-actin aggregation occurs (marked by yellow arrows). (**E**) 3D surface rendering of Caveolin-1, Caveolin-2, and F-actin at downstream end of one flow-aligned HAEC. (**F**) Representative confocal images of Caveolin-1 protein expression for HAECs in low-flow and high-flow regions with quantification of Caveolin-1 cluster index (arbitrary units). Graph represents the average value ± SD from n = 3 biological replicates and statistics calculated using one-tailed, unpaired *t* test; $^{*}P < 0.05$.

Supplemental Figure 3

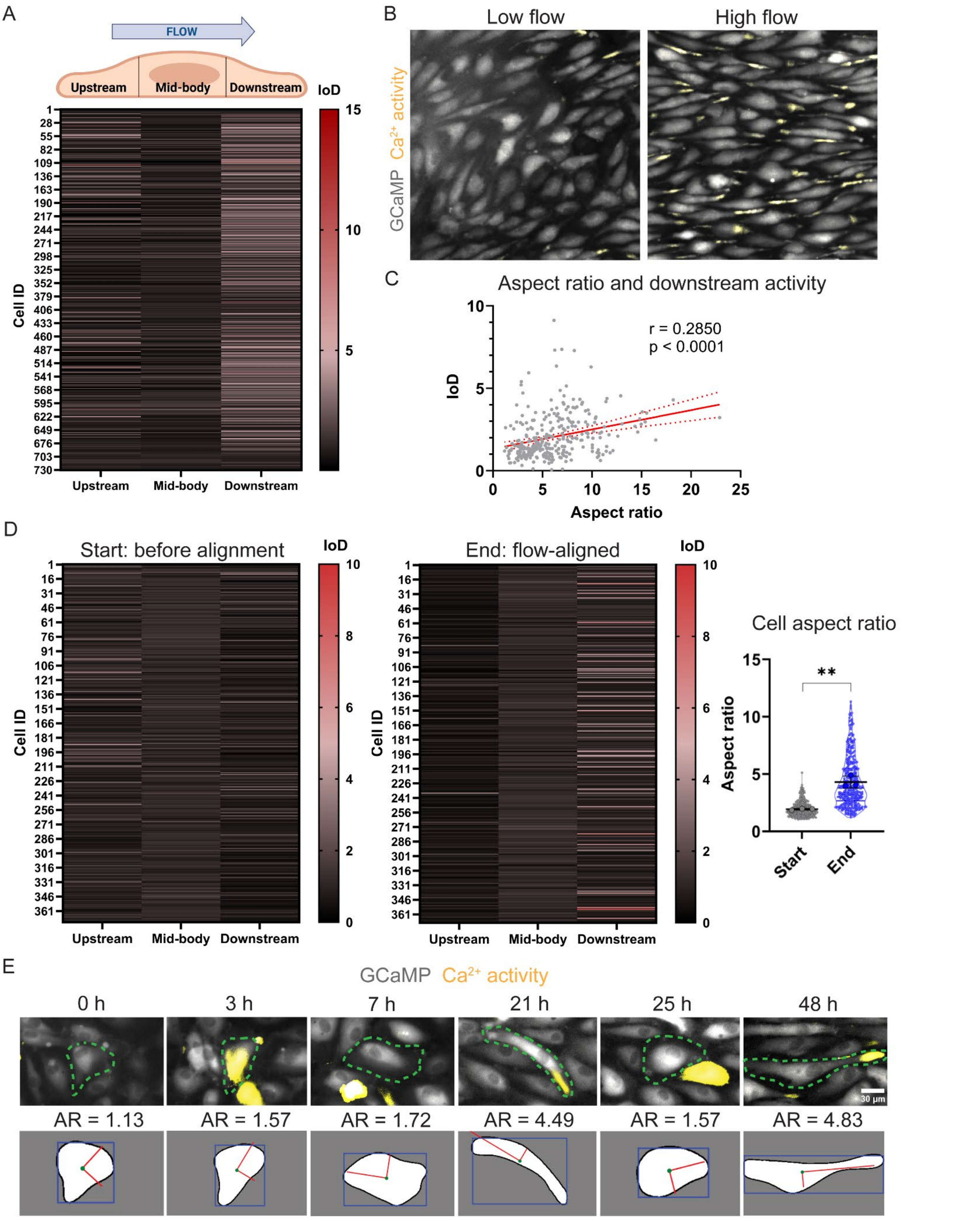

Supplemental Figure 3: Ca^{2+} activity analysis in response to endothelial cell alignment with flow.

(**A**) Heat map displaying subcellular location of Ca^{2+} activity, plotted as the index of dispersion (IoD), for HAECs exposed to high laminar flow (20 dynes/cm^2) and analyzed in Figure 2C. (**B**) Higher magnification images of cells in the low-flow and high-flow regions of the y-shaped slide in Figure 3B where Ca^{2+} spikes are indicated in yellow. (**C**) Correlation analysis of downstream end Ca^{2+} activity and cell aspect ratio. Pearson correlation coefficient (r) is used. (**D**) IoD heat maps displaying the subcellular location of Ca^{2+} activity for HAECs at the start (t = 3 h) of flow (20 dynes/cm^2) and after flow-alignment (t = 48 h) for n = 3 biological replicates. Graph displays the aspect ratios for cells from the same monolayers. IoD and aspect ratio was quantified for full-length cells in the field of view at the start of flow (n = 372) and at the end of flow alignment (n = 369) from 3 biological replicates. $^{**}P < 0.01$ by two-tailed, unpaired *t* test. (**E**) Visualization of one cell (outlined in green dashed line) in response to alignment with flow at t = 0, 3, 7, 21, 25, and 48 h. For each time-point, the single frame GCaMP signal is over-laid with a maximum projection of the time differential GCaMP signal indicating Ca^{2+} activity in yellow. The cell shape and calculated aspect ratio (AR) is shown below for each time-point.

Supplemental Figure 4

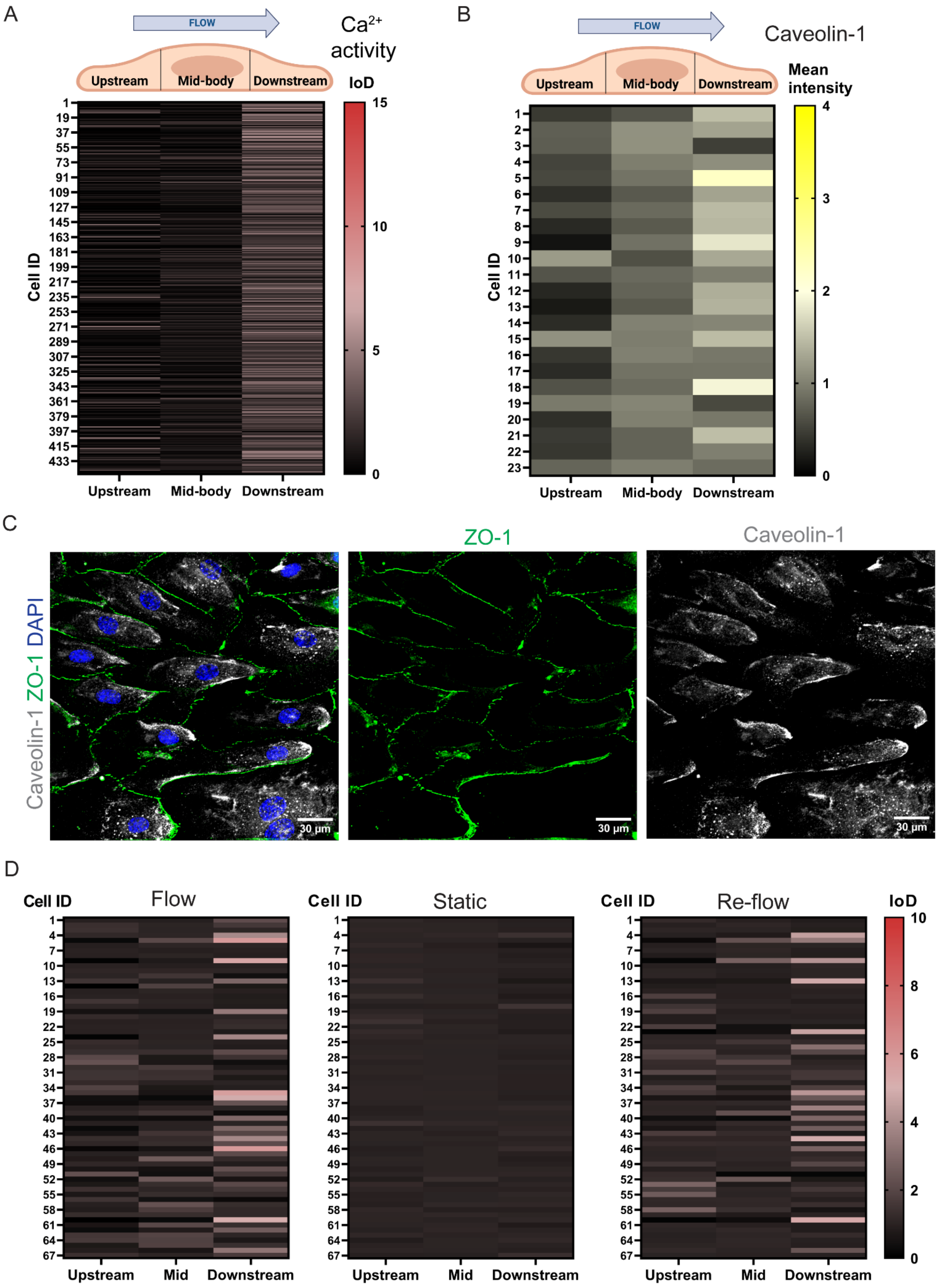

Supplemental Figure 4: Ca^{2+} activity in the presence of high laminar flow for HUVECs and HAECs.

(**A**) IoD values displaying the Ca^{2+} activity for flow-aligned HUVECs. IoD values calculated for 448 segmented cells across n = 3 biological replicates. (**B**) Quantification of subcellular distribution of Caveolin-1 protein for flow-aligned HUVECs (n = 23 cells). (**C**) Representative confocal images for flow-aligned HUVECs stained for Caveolin-1, ZO-1, and DAPI. (**D**) IoD heat maps displaying subcellular location of Ca^{2+} activity for flow-aligned HAECs in the presence of high laminar flow (20 dynes/cm^2) for 30 min, followed by no flow (0 dynes/cm^2) for 30 min, and again exposed to high laminar flow (20 dynes/cm^2) for 30 min. IoD plots show data from 67 cells across n = 3 biological replicates.

Supplemental Figure 5

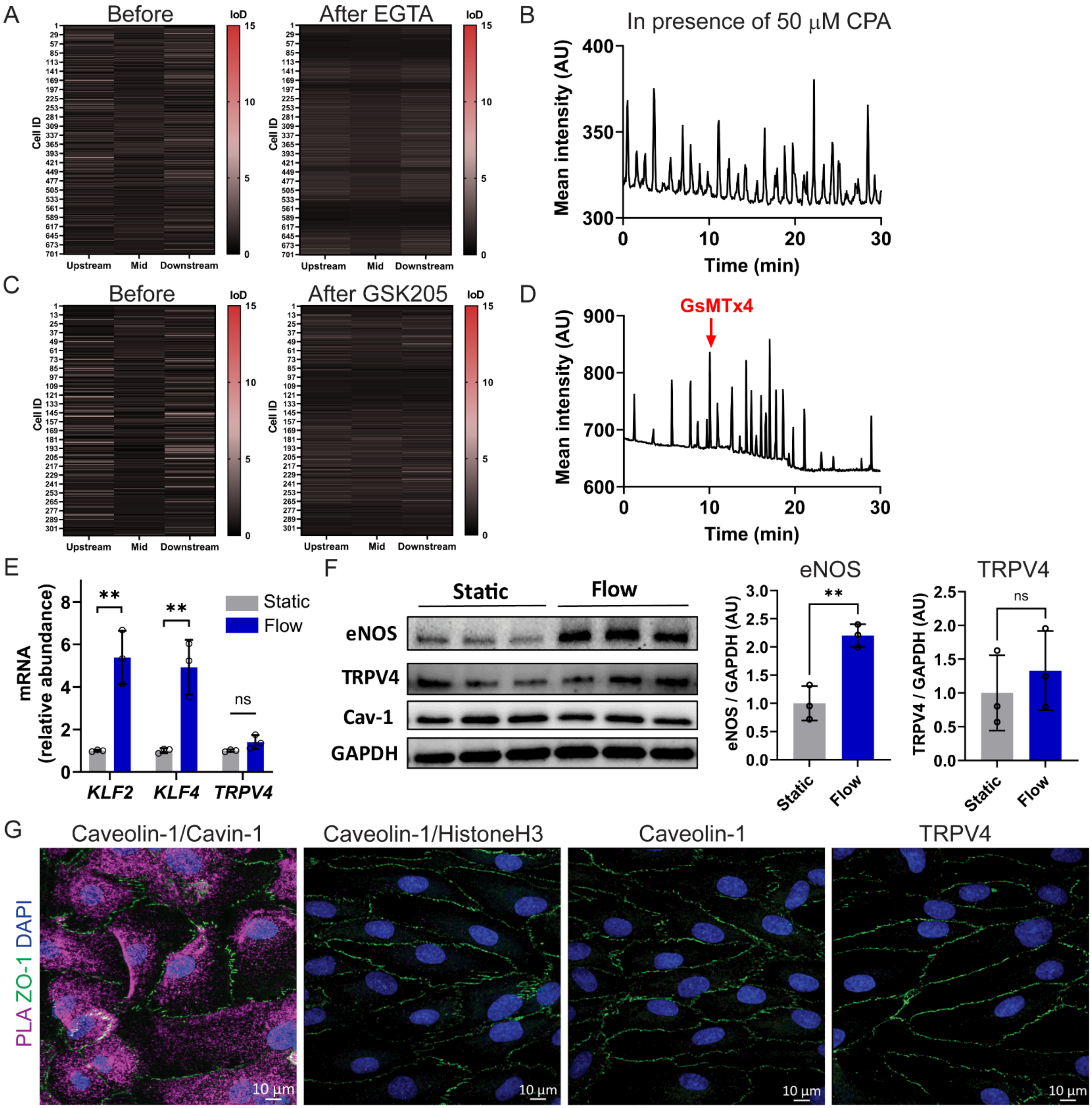

Supplemental Figure 5: TRPV4 expression levels and protein localization under flow.

(**A**) Heat map displaying Ca^{2+} activity for cells in the presence of high laminar flow before and after treatment with EGTA (1.6 mM). (**B**) Representative GCaMP intensity trace showing sustained Ca^{2+} activity at the downstream end in the presence of cyclopiazonic acid (CPA; 50 μM). (**C**) Heat map showing the subcellular location of Ca^{2+} activity for cells under high laminar flow before and after treatment with GSK205 (20 μM). (**D**) Representative GCaMP intensity trace showing Ca^{2+} activity in the presence of high laminar flow before and after the addition of GsMTx4 to the culture media. (**E**) Gene expression analysis in HAECs cultured for 48 h in static or flow conditions shown as mRNA relative abundance, mean ± SD (n = 3 biological replicates per condition). Note the flow-induced expression of *KLF2* and *KLF4* but unchanged expression for *TRPV4*. (**F**) Western blot analysis of eNOS, TRPV4, Caveolin-1 (Cav-1) and GAPDH in HAECs cultured for 48 h under static or flow (20 dynes/cm^2) conditions. Protein expression was determined by densitometry, normalized to GAPDH levels, and then expressed as means ± SD (n = 3 biological replicates per condition). ns, not significant, $^{**}P < 0.001$ by two-tailed, unpaired *t* test. (**G**) Confocal images of representative controls for the PLA. Strong PLA staining was observed for Caveolin-1/Cavin-1. No PLA puncta were observed for Caveolin-1 and nuclear Histone H3 or when Caveolin-1 or TRPV4 antibodies were used on their own.

Supplemental Figure 6

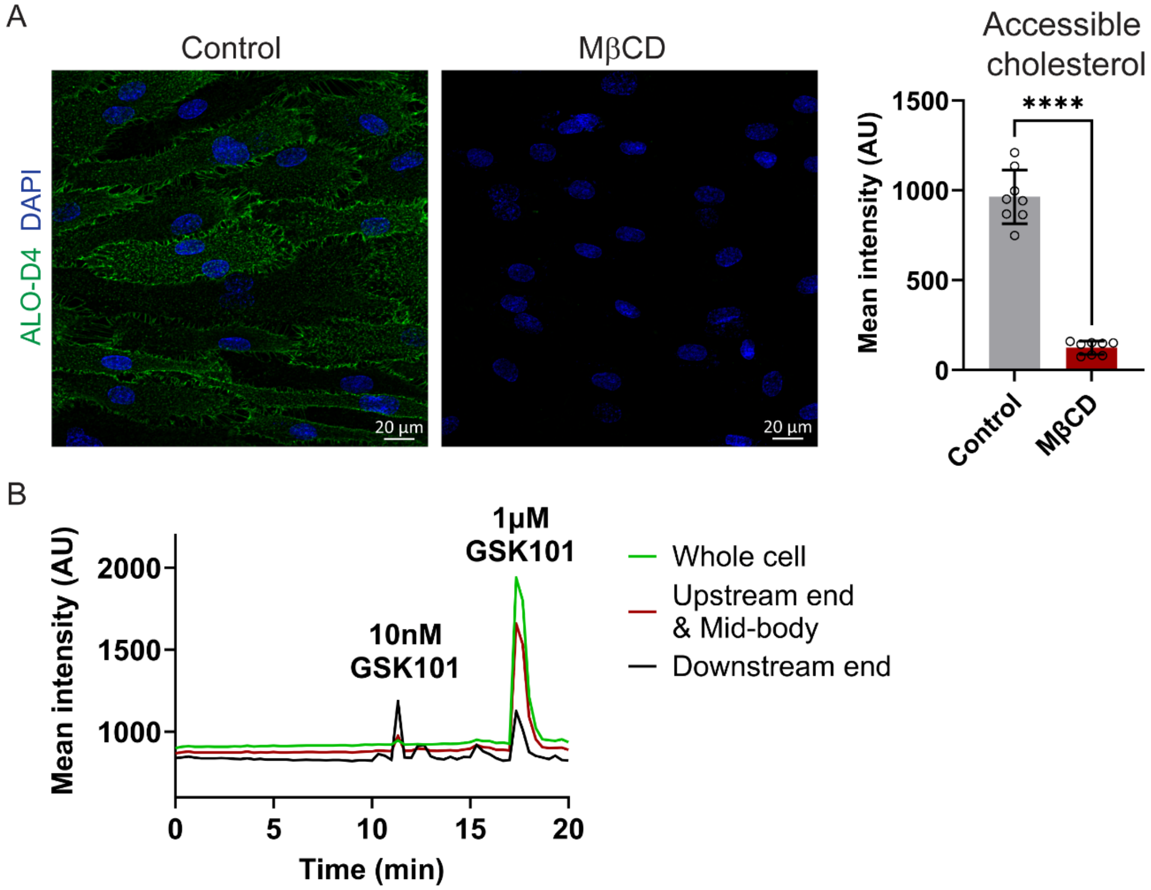

Supplemental Figure 6: Cholesterol depletion using MβCD and induced Ca^{2+} activity.

(**A**) Flow-aligned HAECs were treated with or without MβCD (10 mM) in 1% LPDS statically for 30 min and then subjected to flow (20 dynes/cm^2). After 2 h, cells were subjected to ALOD4-488 (20 μg/mL), fixed and then imaged. Shown are representative confocal images on the left and mean intensity ± SD of the ALO-D4 fluorescent signal from n = 8 replicates per condition. ****$P < 0.0001$ by two-tailed, unpaired *t* test. (**B**) MβCD treated GCaMP HAECs were imaged under flow to record Ca^{2+} activity. No activity was recorded over 10 min. Addition of 10 nM GSK1016790A (GSK101) led to activity at the downstream end. When 1 μM GSK101 was added, activity was observed throughout the entire cell.

Supplemental Figure 7

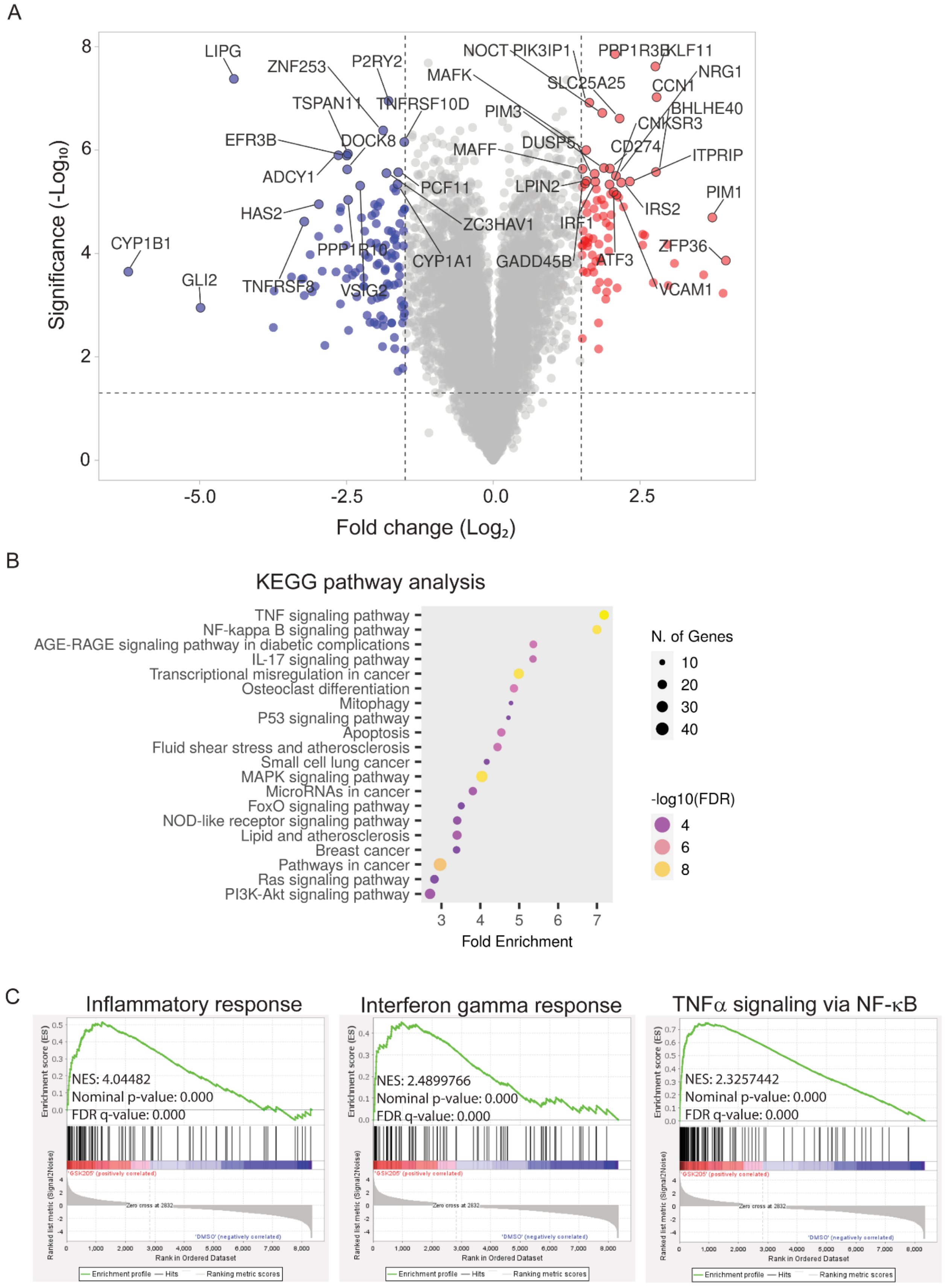

Supplemental Figure 7: Inhibition of TRPV4 activity results in an inflammatory transcriptional fingerprint.

(**A**) Volcano plot of RNASeq data from flow-aligned HAEC monolayers treated with DMSO (control) or TRPV4 antagonist GSK205 (20 μM) for 2 h in the presence of laminar flow (20 dynes/cm^2). Dots, red (increased) and blue (decreased), indicate genes significantly changed more than 2-fold in response to GSK205 treatment. (**B**) KEGG Pathway Analysis of differentially expressed genes shows TNF and NF-κB as top enriched signaling pathways in response to GSK205 treatment. (**C**) Gene Set Enrichment Analysis (GSEA) revealed a significant increase of gene sets involved in the inflammatory response, Interferon-gamma response, and TNFα signaling via NF-κB.

Supplemental Figure 8

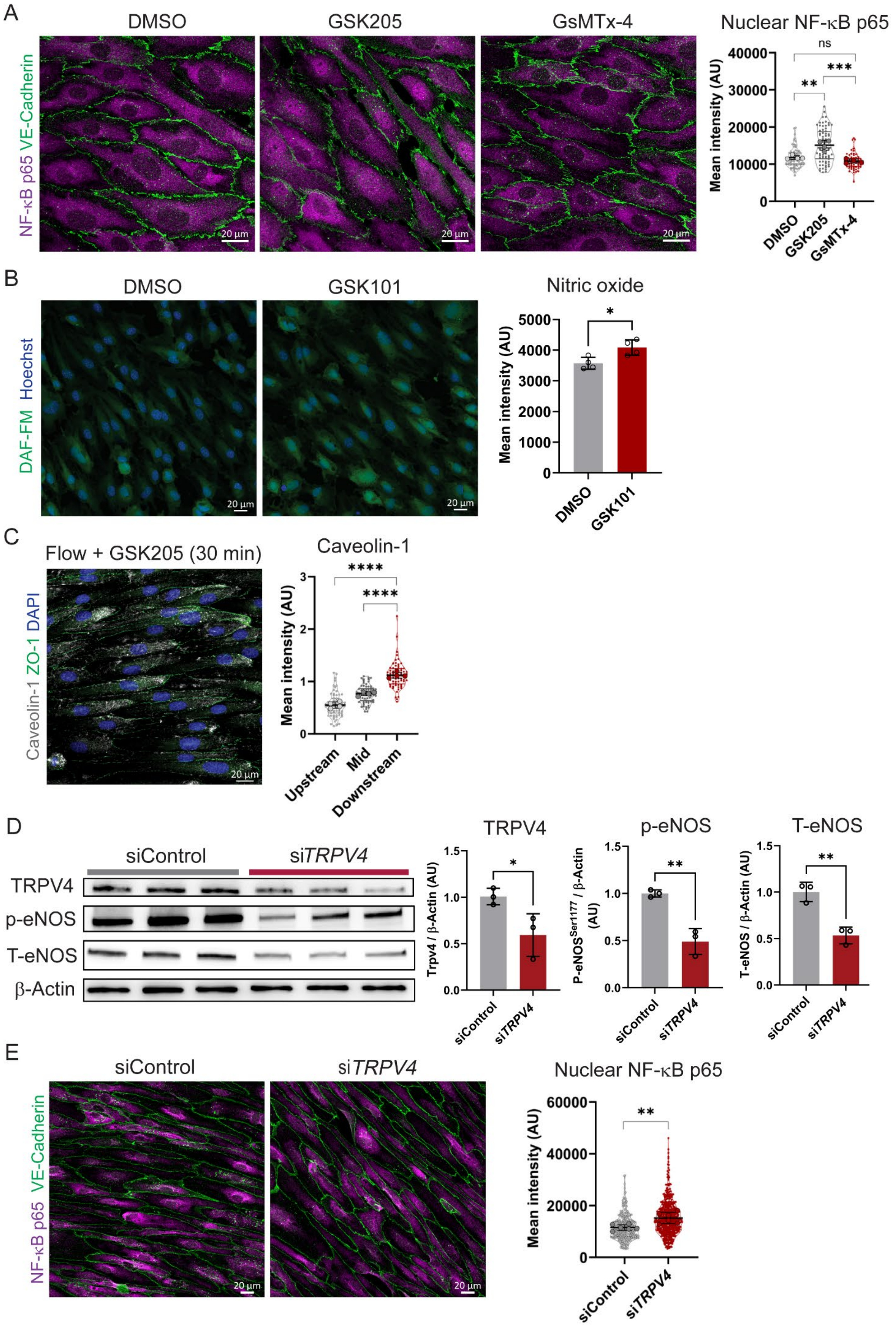

Supplemental Figure 8: TRPV4 protein level and activity affect the production of nitric oxide, reactive oxygen species, and endothelial inflammation.

(**A**) Flow-aligned HAECs were treated with DMSO, GSK205 or GsMTx-4 for 2 h then fixed and stained for NF-κB p65 and VE-Cadherin. Graph represents nuclear fluorescence intensity for 4 biological replicates per group and statistics calculated using one-way ANOVA with post hoc Tukey's multiple comparisons test. (**B**) Flow-aligned HAEC monolayers were treated with DMSO or GSK10116790A (GSK101) for 2 h followed by live cell imaging of DAF-FM and Hoechst. Graph represents DAF-FM mean intensity ± SD (n = 4 biological replicates) and statistics calculated using two-tailed, unpaired *t* test. (**C**) Flow-aligned HAECs were treated with TRPV4 antagonist GSK205 (20 μM) in the presence of laminar flow (20 dynes/cm^2) for 30 min. After 30 min, monolayers were stained for Caveolin-1, ZO-1 and DAPI. Caveolin-1 intensity was quantified for each subcellular region for n = 67 cells from 4 biological replicates. Graph displays mean ± SD with data analyzed by one-way ANOVA and post hoc Tukey's multiple comparisons test. (**D**) HAECs were transfected with siRNA targeting *TRPV4* or non-targeting siRNA (siControl) then exposed to flow (20 dynes/cm^2) for 48 h. Western blot analysis of TRPV4, phospho-eNOS^{ser1177} (p-eNOS), total eNOS (T-NOS) and β-Actin. Protein expression was determined by densitometry, normalized to β-Actin levels, and expressed as means ± SD (n = 3 biological replicates). Statistics calculated by two-tailed unpaired *t* test. (**E**) siControl and si*TRPV4* HAECs were flow aligned for 48 h then stained for NF-κB p65, VE-Cadherin, and DAPI. Nuclear expression of NF-κB p65 is plotted as mean intensity from individual cells across n = 6 biological replicates per condition. Statistics calculated using two-tailed, unpaired *t* test. ns, not significant; *P < 0.05; **P < 0.01; ***P < 0.001; ****P < 0.0001.

Supplemental Figure 9

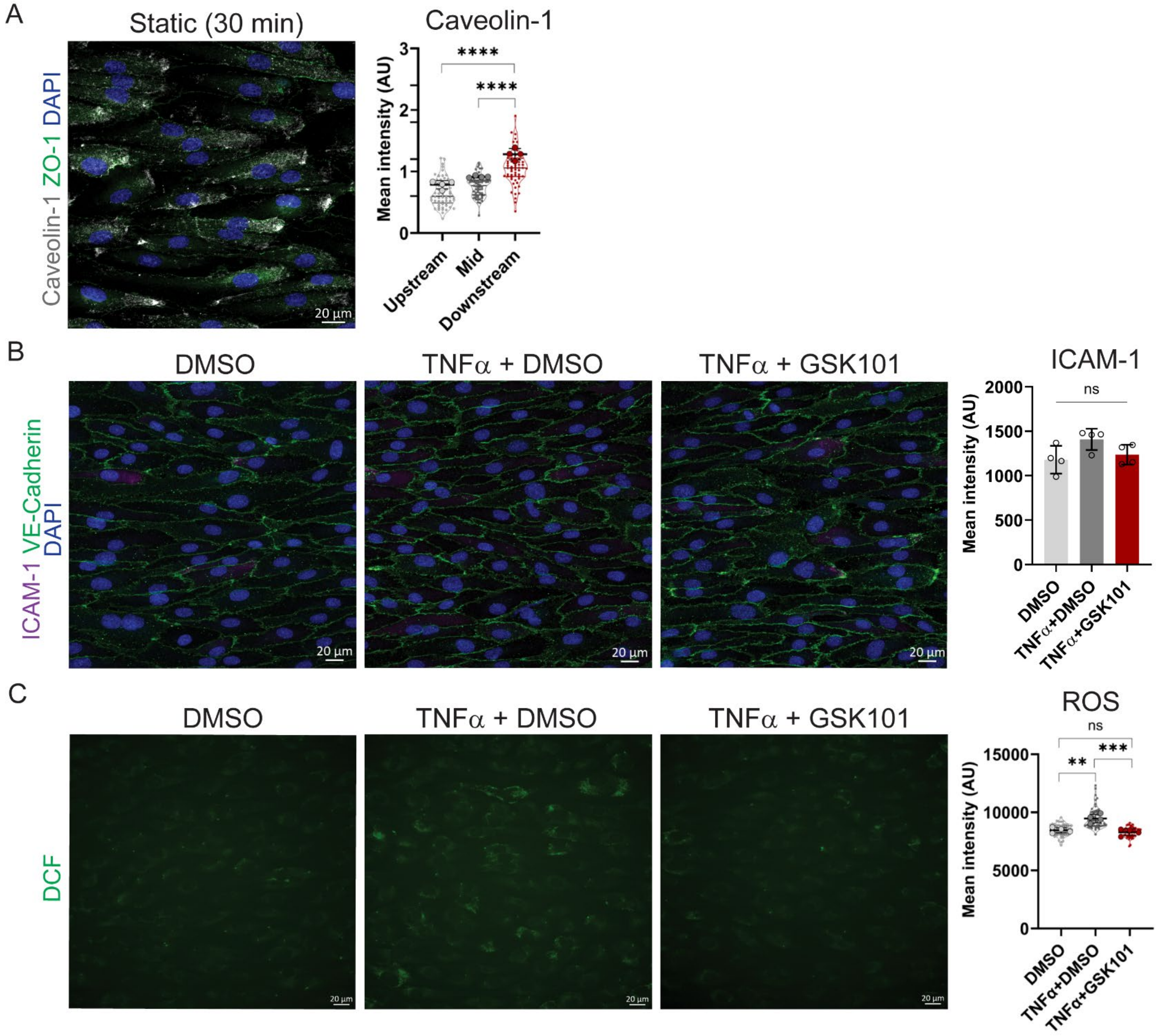

Supplemental Figure 9: Inflammatory response to TNFα.

(**A**) Representative image for flow-aligned HAECs removed from flow for 30 min then fixed and stained. Caveolin-1 staining intensity is quantified for 61 cells from n = 4 biological replicates. Bar graph displays the mean ± SD with data analyzed by one-way ANOVA and post hoc Tukey's multiple comparisons test. ****$P < 0.0001$ (Upstream vs. Downstream; Mid vs. Downstream). (**B**) Flow-aligned HAEC monolayers were treated for 30 min in static conditions with DMSO or TNFα (10 ng/mL) in the presence or absence of GSK101 (10 nM) as in Figure 9D. Monolayers were imaged for junctional marker VE-Cadherin, inflammatory marker ICAM-1, and cell nuclei. ICAM-1 mean fluorescence intensity ± SD (n = 4 biological replicates) did not show statistical difference across the groups. ns, not significant by two-tailed, unpaired *t* test. (**C**) To measure reactive oxygen species (ROS), cells were loaded with CM-H_2DCFDA (5 μM, DCF), then flow aligned for 48 h, followed by 1 h treatment in static conditions with DMSO, 10 ng/mL TNFα + DMSO or 10 ng/mL TNFα + 10 nM GSK101. Mean DCF intensity signal ± SD is shown from 60 cells/condition from 4 biological replicates. Data analyzed by one-way ANOVA and post hoc Tukey's multiple comparisons test; **$P < 0.01$, ***$P < 0.001$ or not significant (ns).

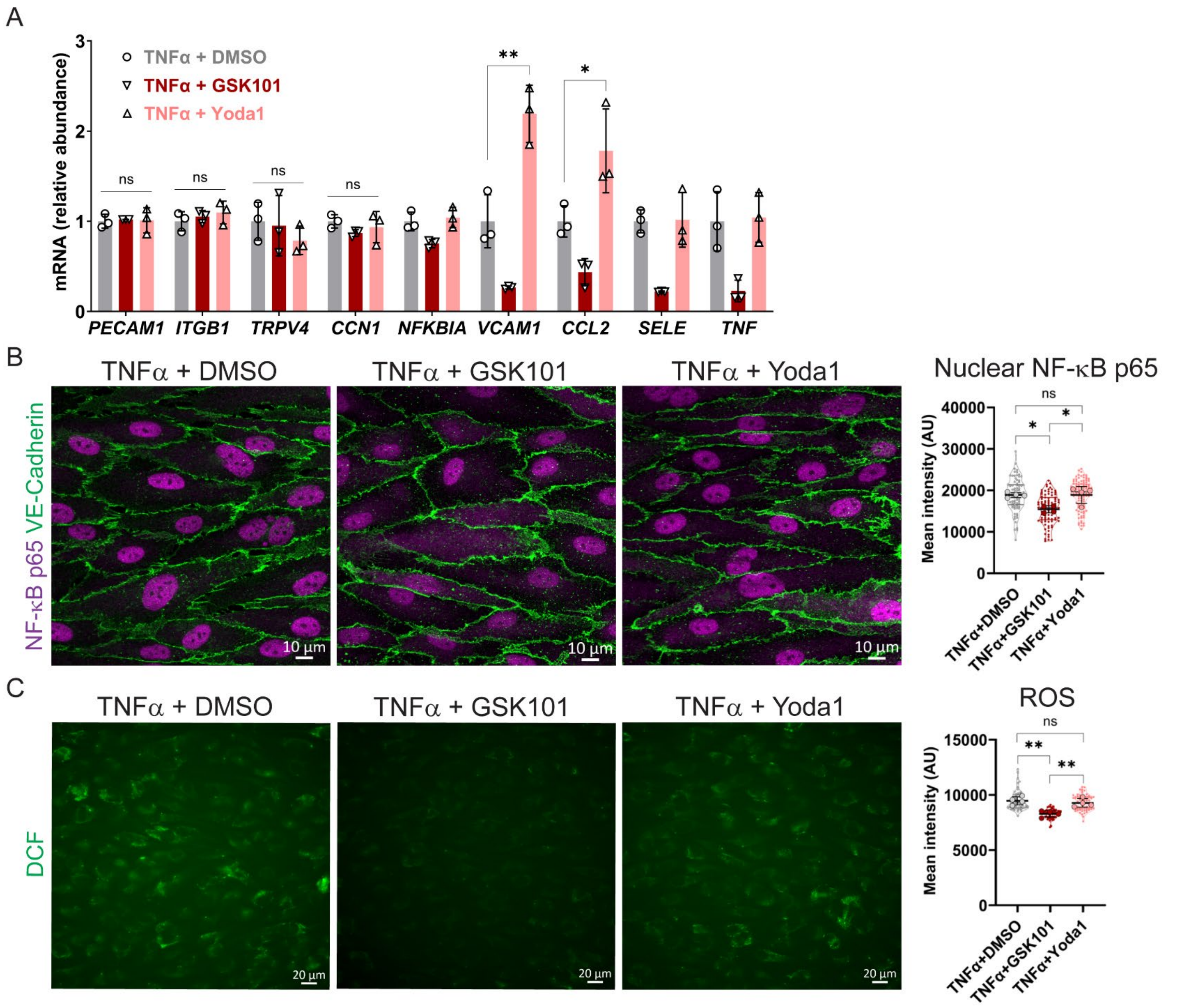
A
TNFα + DMSO
TNFα + GSK101
TNFα + Yoda1
mRNA (relative abundance)
PECAM1
ITGB1
TRPV4
CCN1
NFKBIA
VCAM1
CCL2
SELE
TNF
ns
**
*
B
TNFα + DMSO
TNFα + GSK101
TNFα + Yoda1
NF-κB p65 VE-Cadherin
10 μm
Nuclear NF-κB p65
Mean intensity (AU)
TNFα+DMSO
TNFα+GSK101
TNFα+Yoda1
C
TNFα + DMSO
TNFα + GSK101
TNFα + Yoda1
DCF
20 μm
ROS
Mean intensity (AU)
TNFα+DMSO
TNFα+GSK101
TNFα+Yoda1

Supplemental Figure 10: Activating PIEZO1 does not attenuate the inflammatory response to TNFα.

(**A**) Gene expression was measured by qPCR for TNFα treated monolayers in the presence of DMSO or GSK101 or Yoda1 (n = 3 biological replicates per condition). Gene expression plotted as mean ± SD. $^{*}P < 0.05$, $^{**}P < 0.01$ by two-tailed, unpaired *t* test. Data for TNFα+DMSO and TNFα+GSK101 from Figure 9B. (**B**) Flow-aligned HAEC monolayers were treated with TNFα (10 ng/mL) in the presence of DMSO, GSK101 (10 nM) or Yoda1 (0.5 μM). Following 30 min treatment, monolayers were fixed and stained for DAPI, VE-Cadherin and NF-κB p65. Graph represents mean fluorescence intensity in the nucleus from individual cells per group and statistics calculated using one-way ANOVA with post hoc Tukey's multiple comparisons test; ns, not significant; $^{*}P < 0.05$. (**C**) Quantification of ROS was performed by loading cells with CM-H_2DCFDA (5 μM, DCF), then flow aligned for 48 h, followed by 1 h treatment in static conditions with DMSO, 10 ng/mL TNFα + DMSO or 10 ng/mL TNFα + 10 nM GSK101 or TNFα + 0.5 μM Yoda1. Mean DCF intensity signal ± SD is shown from 60 cells per condition from n =4 biological replicates. Data analyzed by one-way ANOVA and post hoc Tukey's multiple comparisons test; $^{**}P < 0.01$ or not significant (ns). Data for TNFα+DMSO and TNFα+GSK101 is previously presented in Supplemental Figure 9C.

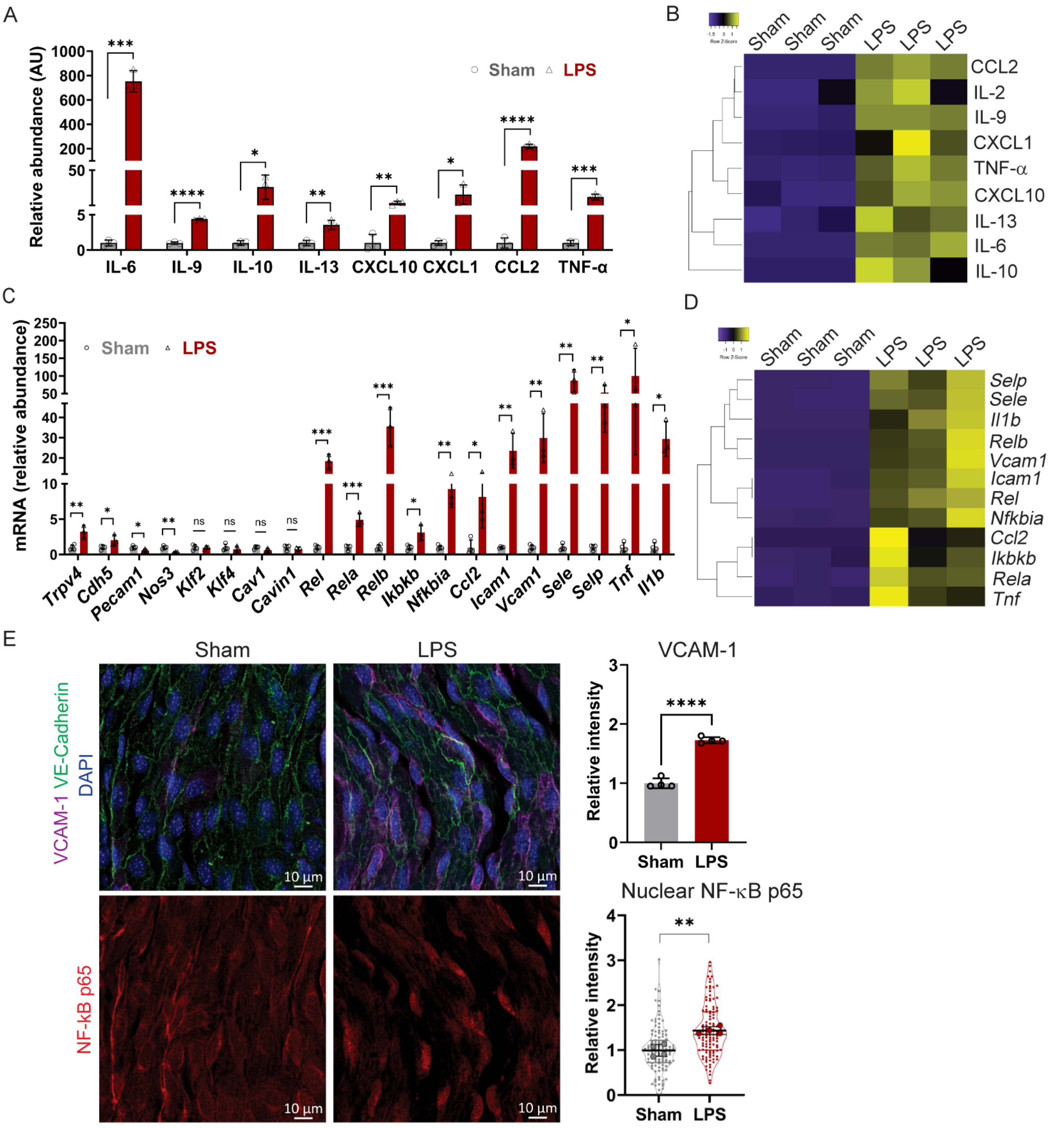
A
Relative abundance (AU)
Sham LPS
IL-6 IL-9 IL-10 IL-13 CXCL10 CXCL1 CCL2 TNF-α
B
Sham Sham Sham LPS LPS LPS
CCL2
IL-2
IL-9
CXCL1
TNF-α
CXCL10
IL-13
IL-6
IL-10
C
mRNA (relative abundance)
Sham LPS
Trpv4 Cdh5 Pecam1 Nos3 Klf2 Klf4 Cav1 Cavin1 Rel Rela Relb Ikbkb Nfkbia Ccl2 Icam1 Vcam1 Sele Selp Tnf Il1b
D
Sham Sham Sham LPS LPS LPS
Selp
Sele
Il1b
Relb
Vcam1
Icam1
Rel
Nfkbia
Ccl2
Ikbkb
Rela
Tnf
E
Sham
LPS
VCAM-1 VE-Cadherin DAPI
NF-kB p65
10 μm
VCAM-1
Relative intensity
Sham LPS
Nuclear NF-κB p65
Relative intensity
Sham LPS

Supplemental Figure 11: LPS injection in vivo results in inflammation.

(**A**) Systemic cytokine expression for sham (DMSO) and LPS treated mice after 4 h. (**B**) As in (A), heat map of plasma cytokine expression. (**C**) Gene expression of aortic endothelium quantified by qPCR of mRNA isolated from the descending aorta of mice injected with sham or LPS. (**D**) Aortic endothelial gene expression plotted as heat map for inflammatory genes measured by qPCR for sham and LPS treated mice. (**E**) En face staining of abdominal aortas from mice injected with sham or LPS. Shown are representative images and associated quantification of VCAM-1 staining intensity and nuclear NF-κB p65 intensity. Graphs represent mean intensities ± SD from n = 4 mice and statistics calculated using two-tailed, unpaired *t* test; ****$P < 0.0001$, **$P < 0.01$.

Supplemental Videos

For all videos, flow direction is left to right.

Video 1. 30 min time-lapse recording of GCaMP expressing flow-aligned HAEC monolayer in the presence of high laminar flow (20 dynes/cm^2).

Video 2. 15 min time-lapse recording of GCaMP expressing flow-aligned HAECs in the presence of high laminar flow (20 dynes/cm^2). Note, the cell outlined is used for analysis performed in Figure 2B.

Video 3. 15 min time-lapse recording of GCaMP expressing flow-aligned HAEC outlined for downstream end (black) and whole cell (green). Note that GCaMP fluorescence signal is displayed as Gold Lookup Table (LUT). Analysis of subcellular Ca^{2+} activity is presented in Figure 2B.

Video 4. 15 min time-lapse recording of GCaMP expressing HAECs in the presence of low flow (~5 dynes/cm^2).

Video 5. 15 min time-lapse recording of GCaMP expressing HAECs in the presence of high flow (~20 dynes/cm^2).

Video 6. 25 min time-lapse recording of GCaMP HAECs in flow convergence region of increasing shear stress from 5 dynes/cm^2 to 20 dynes/cm^2. Analysis of Ca^{2+} activity is presented in Figure 3B.

Video 7. Time-lapse imaging of GCaMP HAECs in response to flow (20 dynes/cm^2) from 0 to 48 h. At each hour, a 10 min time-lapse recording was acquired. Analysis of Ca^{2+} activity before and after flow-alignment is presented in Supplemental Figure 3D.

Video 8. Time-lapse imaging of GCaMP HAECs in response to flow (20 dynes/cm^2) from 0 to 48 h. At each hour, a 10 min time-lapse recording was acquired. Note one cell is highlighted by magnifying lens to capture cell shape at recording timepoints t = 0 h, 3 h, 7 h, 21 h, 25 h and 48 h. Analysis of Ca^{2+} activity and cell aspect ratio is presented in Supplemental Figure 3E.

Video 9. 30 min time-lapse imaging of flow-aligned GCaMP HUVECs in the presence of high laminar flow (20 dynes/cm^2). Analysis of Ca^{2+} activity is presented in Supplemental Figure 4A.

Video 10. 90 min time-lapse imaging of flow-aligned GCaMP HAEC exposed to 30 min laminar flow (20 dynes/cm^2), followed by 30 min static (0 dynes/cm^2) condition and then 30 min laminar flow (20 dynes/cm^2). Note that GCaMP fluorescence signal is displayed as Gold Lookup Table (LUT). Analysis of Ca^{2+} activity at the downstream end is presented in Figure 3C.

Video 11. 10 min time-lapse imaging of GCaMP HAECs seeded on line-patterned chamber recorded in static conditions. GCaMP intensity trace for Ca^{2+} activity is presented in Figure 4D.

Video 12. 30 min time-lapse recording of cholesterol depleted flow-aligned GCaMP HAECs in the presence of high flow (20 dynes/cm^2). At 20 min, the TRPV4 agonist GSK10116790A (GSK101; 10 nM) is added to the culture flowing media. Ca^{2+} activity analysis is displayed in Figure 6 D–F.
