# Supplementary material for: Mechanosensitive membrane domains regulate calcium entry in arterial endothelial cells to protect against inflammation
Source: J Clin Invest. 2024 May 21;134(13):e175057. doi: 10.1172/JCI175057 (PMC11213468; doi:10.1172/JCI175057)
Supplement: Unedited blot and gel images [file jci-134-175057-s267.pdf]

Supplemental Figure 5F

eNOS (133 kDa)

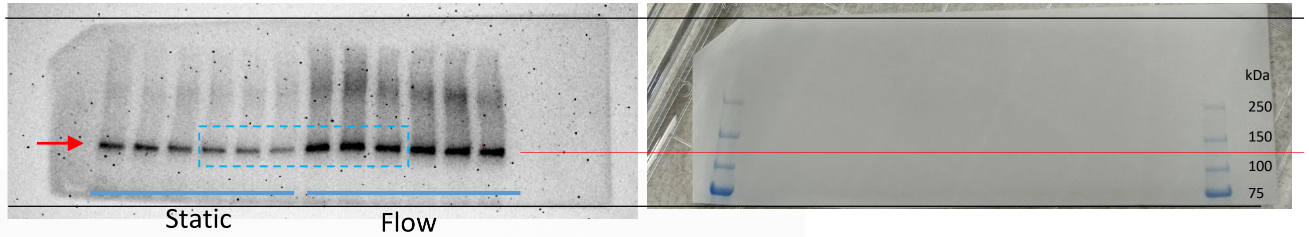

(Santa Cruz, #sc-376751, 1:500, mouse)

TRPV4 (98 kDa)

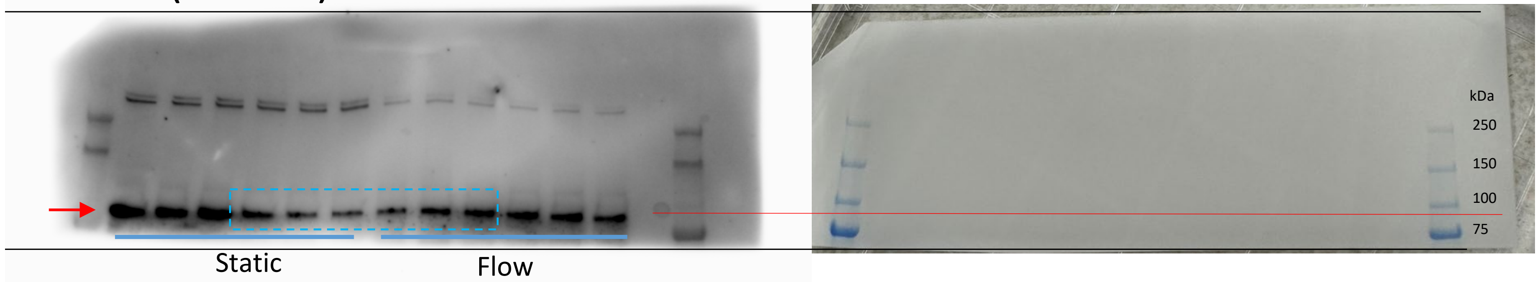

(Alomone Labs, #ACC-034, 1:500, rabbit)

Cav1 (22 kDa)

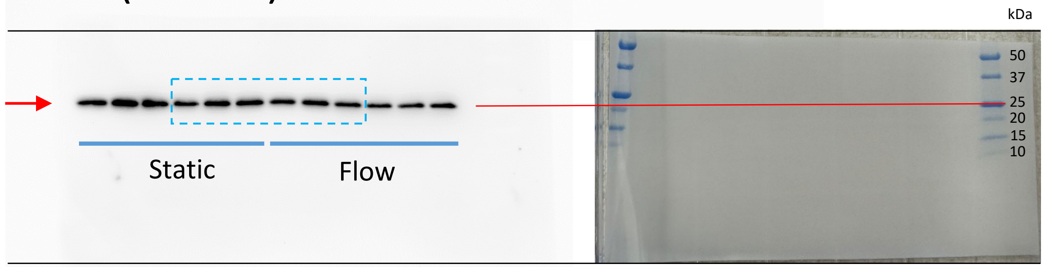

(Santa Cruz, #sc-70516, 1:200, mouse)

GAPDH (36 kDa)

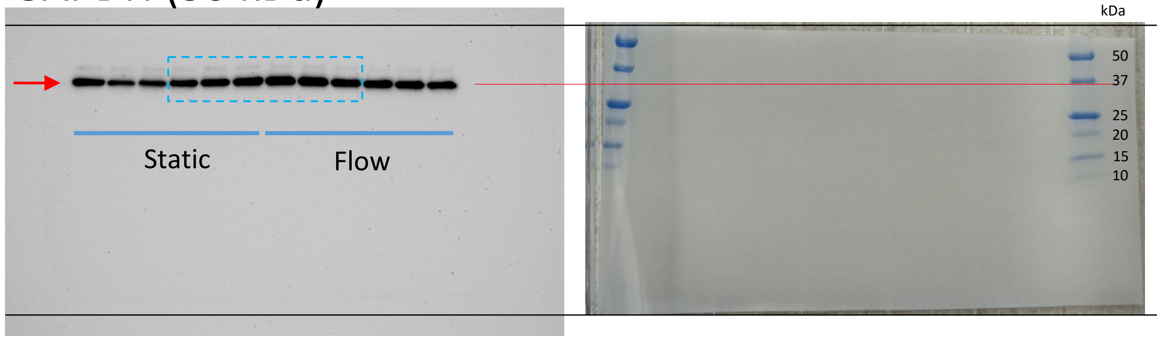

(Cell Signaling Technology, #2118s, 1:1000, rabbit)

Supplemental Figure 8D

T-eNOS (133 kDa)

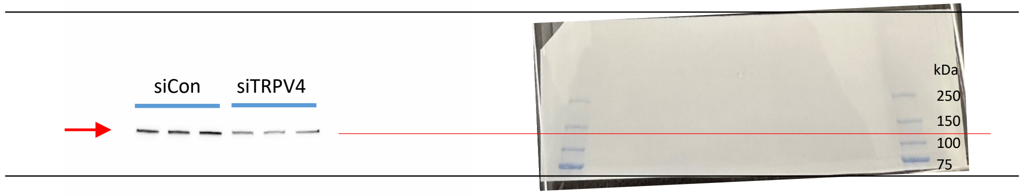

(Santa Cruz, #sc-376751, 1:500, mouse)

TRPV4 (98 kDa)

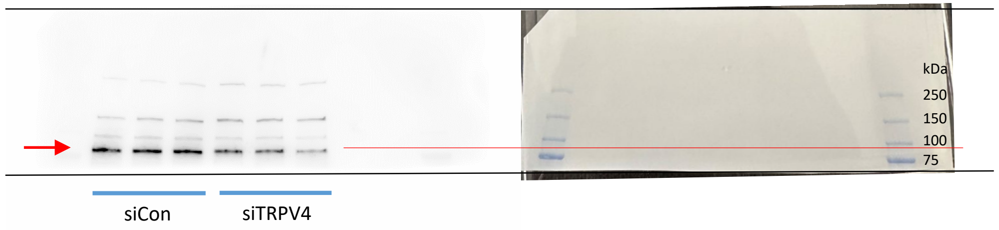

(Alomone Labs, #ACC-034, 1:500, rabbit)

p-eNOS Ser1177 (133 kDa)

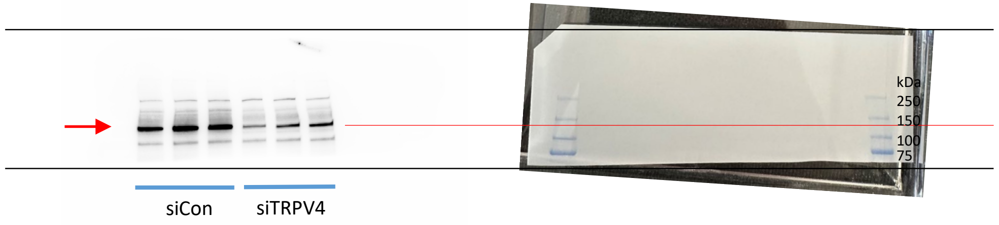

(Invitrogen, #PA5-35879, 1:1000, rabbit)

β-Actin (42 kDa)

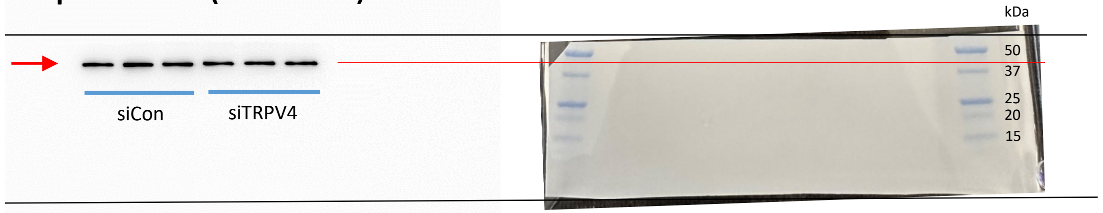

(Sigma, #A5316, 1:1000, mouse)
